# Metabolic Implications of Using BioOrthogonal Non-Canonical Amino Acid Tagging (BONCAT) for Tracking Protein Synthesis

**DOI:** 10.3389/fmicb.2020.00197

**Published:** 2020-02-13

**Authors:** Katherine F. Steward, Brian Eilers, Brian Tripet, Amanda Fuchs, Michael Dorle, Rachel Rawle, Berliza Soriano, Narayanaganesh Balasubramanian, Valérie Copié, Brian Bothner, Roland Hatzenpichler

**Affiliations:** ^1^Department of Chemistry and Biochemistry, Montana State University, Bozeman, MT, United States; ^2^Thermal Biology Institute, Montana State University, Bozeman, MT, United States; ^3^Center for Biofilm Engineering, Montana State University, Bozeman, MT, United States

**Keywords:** metabolomics, BONCAT, non-canonical amino acids, *L*-azidohomoalanine, *L*-homopropargylglycine

## Abstract

BioOrthogonal Non-Canonical Amino acid Tagging (BONCAT) is a powerful tool for tracking protein synthesis on the level of single cells within communities and whole organisms. A basic premise of BONCAT is that the non-canonical amino acids (NCAA) used to track translational activity do not significantly alter cellular physiology. If the NCAA would induce changes in the metabolic state of cells, interpretation of BONCAT studies could be challenging. To address this knowledge-gap, we have used a global metabolomics analyses to assess the intracellular effects of NCAA incorporation. Two NCAA were tested: *L*-azidohomoalanine (AHA) and *L*-homopropargylglycine (HPG); *L*-methionine (MET) was used as a minimal stress baseline control. Liquid Chromatography Mass Spectrometry (LC-MS) and Nuclear Magnetic Resonance (NMR) were used to characterize intracellular metabolite profiles of *Escherichia coli* cultures, with multivariate statistical analysis using XCMS and MetaboAnalyst. Results show that doping with NCAA induces metabolic changes, however, the metabolic impact was not dramatic. A second set of experiments in which cultures were placed under mild stress to simulate real-world environmental conditions showed a more consistent and more robust perturbation. Pathways that changed include amino acid and protein synthesis, choline and betaine, and the TCA cycle. Globally, these changes were statistically minor, indicating that NCAA are unlikely to exert a significant impact on cells during incorporation. Our results are consistent with previous reports of NCAA doping under replete conditions and extend these results to bacterial growth under environmentally relevant conditions. Our work highlights the power of metabolomics studies in detecting cellular response to growth conditions and the complementarity of NMR and LCMS as omics tools.

## Introduction

Dieterich et al. (2006) introduced a method for visualizing newly synthesized proteins in mammalian cells termed BioOrthogonal Non-Canonical Amino acid Tagging (BONCAT). BONCAT facilitates the tracking and localization of protein translation in single cells following a short incubation with a synthetic amino acid that later can be detected via azide-alkyne click-chemistry, a sensitive and precise biocompatible reaction (Kolb et al., 2001). BONCAT has proven to be particularly useful for monitoring cellular activity in complex microbial communities (Hatzenpichler et al., 2014, 2016; Samo et al., 2014; Leizeaga et al., 2017; Sebastián et al., 2019), and adds a convenient approach to the molecular tool box available for analyzing microbial community function (Hatzenpichler et al., 2020) because it avoids the use of radioactive substrates and is understood to only minimally impact protein structure and cell physiology. Currently, the two most widely used non-canonical amino acids (NCAA) are *L*-azidohomoalanine (AHA) and *L*-homopropargylglycine (HPG), which both replace *L*-methionine (MET) during translation (Kiick et al., 2002). These amino acids contain either an azide (AHA) or an alkyne functional group (HPG) which are amenable to azide-alkyne click chemistry (Kolb et al., 2001). Experimental protocols for performing BONCAT studies and click-labeling newly made proteins are well established in microbiology and microbial ecology (Bagert et al., 2014; Hatzenpichler et al., 2014, 2016; Mahdavi et al., 2014; Hatzenpichler and Orphan, 2015; Babin M. B. et al., 2016; Bagert et al., 2016).

Phenotypic markers of optical density, behavioral tests, and responses to visual cues have been utilized to assess the impact of cell treatments with NCAA. Studies on HeLa cells (Bagert et al., 2014), a range of bacterial and archaeal pure cultures (Bagert et al., 2014; Hatzenpichler et al., 2014; Hatzenpichler and Orphan, 2015), and environmental samples (Hatzenpichler et al., 2014, 2016) have demonstrated that the addition of low concentrations (nM-mM range) of AHA or HPG to a sample over short periods of time (typically 1–2 cell generations) has only minimal effects on the physiology, growth rate, or protein expression patterns of organisms. Hinz et al. studied zebrafish and investigated the potential effects of NCAA labeling *in vivo*, which revealed that AHA was successfully incorporated into proteins in a ratio consistent with time and concentration, that AHA was non-toxic and had no detrimental effect on animal behavior (Hinz et al., 2012). A recent proteomic study investigating the effect of AHA and HPG on protein expression and the ability to incorporate these reagents into mice showed that a small percentage (∼10%) of proteins change their expression patterns in response to AHA doping (Calve et al., 2016). Lastly, a recent study indicated that the incorporation of AHA into a model protein only minimally affected the protein tertiary structure (Lehner et al., 2017). The recent application of proteomics investigating the cell machinery have also shown that AHA and HPG have little impact on the overall fitness of the organism (Dieterich et al., 2006; Landgraf et al., 2015). However, a deeper look into the metabolism of NCAA doped organisms has, to our knowledge, never been carried out.

This study aimed to characterize the metabolome of *Escherichia coli* when grown in the presence of NCAA and identify potentially differentiated metabolite patterns that might inform us on the metabolic impact of NCAA on cell homeostasis and organismal health. In order to investigate how NCAA exposure affects intracellular metabolism, *E. coli* cell cultures were grown with and without AHA or HPG. One sample group included additional MET as a minimal perturbation to be used as a control experiment for baseline stress due to media supplementation. Control cultures were also grown in media without amino acid amendment. “Real world” experimental and cell culturing conditions were utilized in our analysis to best evaluate the potential effects of NCAA, and to mimic current field work in the environment that attempts to find suitable growth conditions for otherwise unculturable microorganisms. Comprehensive metabolite mapping techniques using Liquid Chromatography Mass Spectrometry (LC-MS) and Nuclear Magnetic Resonance (NMR) spectroscopy were employed to assess potential metabolome differences between different *E. coli* cell cultures and growth conditions.

## Materials and Methods

### Reagents

HPLC grade solvents: water, methanol and acetonitrile were purchased from Fisher (Waltham, MA, United States). AHA and HPG were purchased from Click Chemistry Tools (Scottsdale, AZ, United States). All other chemicals were purchased from Millipore Sigma (St. Louis, MO, United States) and were used as provided, with no additional purification steps.

### Cell Culturing

An overnight culture of *E. coli* K12 DH10B, which had been grown on M9 minimal medium (200 mg/L thiamine, 0.2% glucose), was inoculated 1:20 into 6 L of M9 medium (200 mg/L thiamine, 0.2% glucose) to yield a fresh culture of optical density, measured at a wavelength l of 600 nm, i.e., OD_600_ of 0.041. 150 mL aliquots of this culture were then aliquoted into 36 Erlenmeyer flasks, which were incubated at 37°C on rotary shakers run at 200 rpm. Temperatures were independently checked with a thermometer at regular intervals to validate that temperatures were consistent across incubators throughout the experiments. Immediately following inoculation, the following incubations were started: five flasks each for (1) 50 μM MET; (2) 50 μM AHA; and (3) 50 μM HPG. One additional control flask without amendment was used to monitor growth of the cultures via optical density (OD_600_). This was done to avoid disturbing the experimental cultures given the large number of flasks. Growth experiments with 50 μM amino acid addition (MET, AHA, or HPG) were stopped after 85 min of incubation when the control flask had reached an OD_600_ of 0.072, corresponding to ∼0.74 cell generations (Supplementary Figure S1). The 2 × 50 mL cell cultures were then decanted into two 50 mL tubes. Tubes were centrifuged for 5 min at 4,700 *g* at room temperature. Resulting supernatants were decanted and the cell pellets flash-frozen in liquid N_2_ and stored at −80°C until further processing. After these samples had been stored at −80°C, the remaining 20 flasks were processed the following way: 1 mM of (1) MET, (2) AHA, and (3) HPG were added to 5 flasks; Five additional culture flasks served as no-amendment control, which were used to track cell growth. The incubation was continued as described, with a starting OD_600_ of control cell cultures of 0.27, and stopped after 5 min of amino acid pulse labeling whereby the control cultures had reached an OD_600_ of 0.31, corresponding to ∼0.04 cell generations (Supplementary Table S1). Cells were pelleted, pellets flash frozen, and samples stored as described above. Cultures for the heat stress experiments were conducted as described above except that cell cultures were grown and maintained at 42°C.

### Metabolite Extraction

*Escherichia coli* intracellular metabolites were extracted using published protocols (Hamerly et al., 2015). Briefly, frozen cell pellets were re-suspended with water, then sonicated using a Biologics Ultrasonic Homogenizer model 3000 for 10 pulses of 3 s each. Resulting supernatant was centrifuged and transferred to 10 mL scintillation vials to which four volumes of ice cold acetone were added, followed by storage of the samples at −80°C overnight for protein precipitation. Protein concentration in the samples was determined using a Bradford assay (Bradford, 1976) (Supplementary Table S2). Samples were vortexed, centrifuged, and split into two fractions for concurrent analysis by LC-MS and NMR: 1 mL for LC-MS analysis and 4 mL for NMR metabolomics analysis. Both fractions were dried completely using vacuum speed concentration with no heat, and subsequently frozen at −80°C until further use.

### LCMS Instrumentation and Metabolite Analysis

The dried metabolite fraction used for liquid chromatography mass spectrometry (LC-MS) was re-suspended with 20 μL of 50:50 MeOH/H_2_O before injection into the mass spectrometer. MS-based analysis of polar metabolites was accomplished using an Agilent 1290 ultra-high performance liquid chromatography (UPLC) system coupled to an Agilent 6538 Accurate-Mass quadrupole Time of Flight (TOF) mass spectrometer. A Cogent diamond hydride HILIC chromatography column (2.2 μM, 120 A, 150 mm × 2.1 mm Microsolv, Leland, NC, United States) was used for metabolite separation. The gradient began with solvent B (0.1% formic acid in acetonitrile) for 2 min at 50%, followed by a gradient ramp of 50–100% B over 14 min. This step was followed by a hold at 100% solvent B for 1 min, and then return to initial conditions. Mass analysis was conducted in positive mode with a capillary voltage of 3500 V, dry gas temperature of 350°C at a flow of 8 L/min and the nebulizer was set at 60 psi, injecting 2 μL sample volumes, with blanks run intermittently between samples. Data acquisition parameters were as follows: 50–1,000 mass range at 1 Hz scan rate with a resolution of 18,000. Accuracy based on calibration standards was approximately 5 ppm.

### Statistical Analysis of MS Data

Extracted ion chromatograms, peak detection, peak annotation, chromatogram alignment, gap filling and relative quantitation of identified features was completed using MZmine (Pluskal et al., 2010), MetaboAnalyst (Chong and Xia, 2018), and XCMS (Tautenhahn et al., 2012). Metabolite identifications were made based on exact mass and retention time matches to authentic standards using an in house library of ∼500 compounds. Statistical analysis of the MZmine output was done using Microsoft Excel version 2016 and MetaboAnalyst v4.0. XCMS utilizes an all-inclusive processing package with a similar workflow, in which it extracts chromatograms, identifies peaks, matches peaks across samples, gap fills, performs statistical analyses and *in silico* compound identification, and graphical visualization of the data. Identifications of unknown features were made using the MetLin Metabolite Database, which provided a list of possible metabolites based on exact mass, species, and likelihood (Myers et al., 2017).

### Sample Preparation and NMR Analysis

Dried metabolite mixtures were re-suspended in 600 μL of NMR buffer (containing 0.25 mM 4,4-dimethyl-4-silapentane-1-sulfonic acid (DSS) in 90%H_2_O/10% D_2_O, 25 mM sodium phosphate, pH 7), and transferred into 5 mm NMR tubes. All one dimensional (1D) 1H NMR spectra were recorded at 298 K using a Bruker AVANCE III solution NMR spectrometer operating at 600.13 MHz 1H Larmor frequency and equipped with a 5 mm liquid-helium-cooled TCI cryoprobe with Z-gradient and a SampleJetTM automatic sample loading system. 1D 1H NMR data were acquired using the Bruker supplied 1D excitation sculpting water suppression pulse sequence ‘*zgesgp’* with 256 scans, a 1H spectral window of 9,600 Hz, 32K data points, a dwell time interval of 52 μsec, and a recycle delay of 5 s between scan acquisitions. The data were first processed with the Bruker TOPSPIN 3.5 software^1^ using standard parameters for chemical shift referencing using the DSS signal and line broadening (0.3 Hz). Spectral phases were manually adjusted, and a polynomial function was applied (qfil, 0.2 ppm width) on the residual water peak to remove its signal. Metabolite identification and quantification were carried out using the Chenomx^TM^ NMR suite software (version 8.3)^2^ and its associated 600 MHz small molecule reference spectral database. DSS was used as an internal standard for metabolite quantification, while imidazole NMR signals were used to correct for small chemical shift changes arising from slight pH variations between samples. The metabolite concentration tables (mM) generated with Chenomx were exported to a.csv file and converted to μM, and normalized to sample protein concentration as established from Bradford protein assays.

Validation of metabolite IDs, which were annotated in Chenomx^3^, was accomplished using 2D ^1^H-^1^H and 2D ^1^H-^13^C total correlation spectroscopy (TOCSY) NMR or by spiking, when available, pure metabolite standards into the samples and monitoring resulting spectral changes in the 1D ^1^H NMR spectra. 2D ^1^H-^1^H TOCSY spectra were acquired for representative samples using the Bruker-supplied ‘mlevphpr.2/mlevgpph19’ pulse sequences (256 × 2048 data points, 2 s relaxation delay, 32 transients per FID,^1^H spectral window of 6602.11 Hz, 80 ms TOCSY spin lock mixing period). 2D ^1^H-^1^H TOCSY spectra were processed using Topspin software (Bruker version 3.2)^4^.

### Statistical Analysis of NMR Data

The NMR-based metabolite data were uploaded to the MetaboAnalyst v4.0 web server for multivariate statistical analysis. Metabolite concentrations were normalized by log-transformation and auto-scaling (mean centered divided by the standard deviation of each variable) prior to univariate and multivariate statistical analysis. Student *t*-test, principal component analysis (PCA) and partial least squares discriminant analysis (PLS-DA) were performed to identify potentially distinct metabolite patterns between the *E. coli* sample groups grown under the different conditions. Variable importance in projection (VIP) plots generated from the PLS-DA data were employed to assess the importance of each variable (i.e., metabolite) in the projection used in PLS-DA model building; statistics were calculated for the data shown in Figure 5, using 3 components, yielding Q2 and R2 values of 0.646 and 0.913, respectively. PLS-DA model validity was further assessed using the (B/W) permutation test function of MetaboAnalyst which, using 2,000 permutation steps yielded a *p*-value of <5 × e-04, as a measure of the significance of the PLS-DA model. For hierarchical clustering analysis (HCA), distances were measured using a Euclidean correlation and clustering by the Ward algorithm.

## Results and Discussion

### Mass Spectrometry-Based Metabolomics of Non-canonical Amino Acids

**FIGURE 1:**
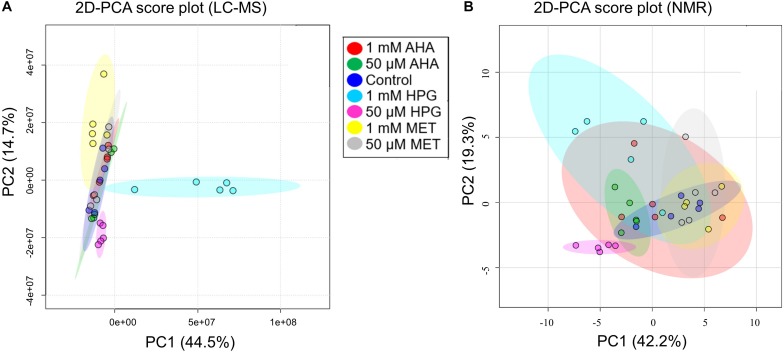
2D-PCA plots of all experimental conditions from *E. coli* NCAA experiment. **(A)** MS data and **(B)** NMR data are shown. **(A)** The PCA plot of the MS data shows that only cultures grown with 1 mM HPG separate from the other experiment conditions. **(B)** The NMR data show significant overlap and a lack of differentiation between experimental groups, the only exception being the group with 50 μM HPG.

An initial set of experiments was conducted to determine the physiological impact of NCAA additions to *E. coli* cell cultures under otherwise normal growth conditions. This study established which NCAA concentrations are needed to evaluate changes in the metabolome of *E. coli* that may be relevant to normal growth conditions (i.e., cells grown at 37°C). Cultures were spiked with either 1 mM or 50 μM concentrations of AHA, HPG, or MET. MET was added as a baseline perturbation control experiment to account for the impact additional amino acid would have on the metabolism of *E. coli*, as opposed to the control group, which had no amendment to the minimal growth medium. Metabolite extracts from the six conditions and control groups were prepared and analyzed by LC-MS using a high-resolution Q-TOF instrument. A total of 4,036 mass features were detected across all sample groups using the MZmine data reduction approach, as described above. Statistical analysis was done in Excel (2016) using the MZmine output, with additional statistical analysis performed using MetaboAnalyst (Chong and Xia, 2018) and XCMS (Tautenhahn et al., 2012). PCA was used to gather information about the variation between sample treatments and replicates. 2D-PCA plots indicated no clear separation among the different experimental groups when all m/z features were analyzed as a single input (Figure 1A). The 1 mM MET and both HPG groups displayed the largest separation from the other treatment groups. While principal component 1 (PC1) accounted for 44.5% of the variance, it primarily separated the 1 mM HPG samples from the other experimental conditions. The second principal component (PC2) accounted for 14.7% of the variance but did little to differentiate between the different sample groups. Overall, there was greater variation between sample treatment replicates than between the different sample treatment groups.

**FIGURE 2:**
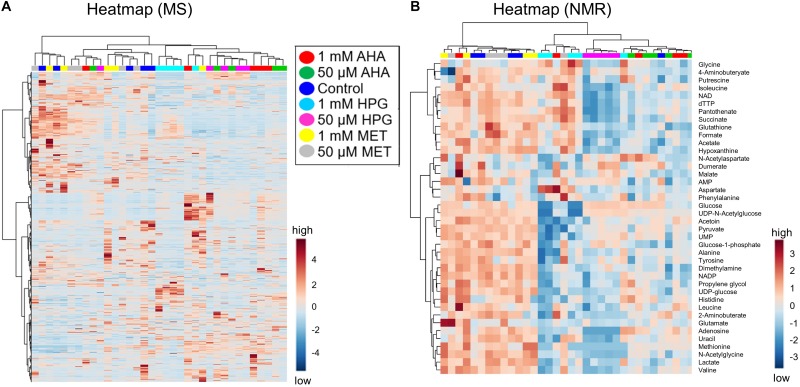
Heatmaps of treatment groups clustered on metabolite intensity from *E. coli* NCAA experiment. **(A)** MS data and **(B)** NMR data are shown. The scale of the heat map indicates blue as lowest and red as highest in abundance as calculated across sample groups after normalization using fold change. **(A)** The heatmap with HCA of 4,036 features as detected by MS is shown. The lack of clustering of the different experimental groups and no significant patterns of up- or down-regulated features for the different groups are indicative of a lack of differentiation between sample types. **(B)** The NMR data (40 metabolites) also shows a lack of group clustering, the only exception being the group with 50 μM HPG. For enlarged images with metabolite names and sample identifications see Supplementary Figure S5.

A heatmap was constructed to visualize differentiated MS features between treatment groups. Heatmaps are a powerful tool for visualizing trends and correlated changes via hierarchical clustering across all samples and all features. The control and baseline-MET samples intermingled, while AHA and the HPG sample treatments mixed on the hierarchal cluster but were generally clustered apart from the MET and control samples (Figure 2A). The heatmap patterns indicated that not all replicates from each treatment clustered with each other; however, a general grouping by type and concentration of NCAA was discernable. The relatively few “hot zones,” or regions of high (dark red) or low (dark blue) abundance features on the heatmap suggested that only a small number of MS features exhibited log2fold changes > 2, indicating that generally minor metabolic differences existed between the different *E. coli* treatment groups. Based on 2D-PCA, ANOVA (Supplementary Table S3), *t*-test and HCA, we thus concluded, from the global MS data, that only small metabolic changes are occurring in *E. coli* grown in the presence of NCAA.

### NMR Metabolite Profiles of *E. coli* Grown in the Presence of Non-canonical Amino Acids

Intracellular metabolite extracts from the same *E. coli* cell cultures were analyzed using 1D ^1^H NMR spectroscopy. As with the MS studies, the NMR metabolomics data readily detected some changes in the metabolome of *E. coli* as a function of NCAA incorporation. However, these metabolite pattern changes were found to be relatively small and insufficient to unambiguously distinguish the different *E. coli* cell cultures based on 2D-PCA and HCA analyses (Figures 1A, 2B) of the different NMR-based metabolite profiles. 54 metabolites were annotated by analysis of the 1D ^1^H NMR spectra of the *E. coli* intracellular metabolite extracts using Chenomx (Supplementary Table S4). These metabolite IDs were further validated using spiking of standards and 2D ^1^H-^1^H and natural abundance ^1^H-^13^C TOCSY experiments (Supplementary Table S5).

Metabolite patterns between the different *E. coli* sample groups, i.e., *E. coli* grown with MET, HPG and AHA at 1 mM and 50 μM conditions, were investigated by PCA analysis of resulting NMR-based metabolite profiles and concentrations (Figure 1B). As with the MS data, no clear clustering pattern or group separation were observable under these experimental growth conditions. The lack of separation along principal components 1 and 2 was particularly noticeable, leading us to conclude that while there exist metabolome differences between the different *E. coli* treatment groups, the NMR-based metabolite profile differences are not sufficiently pronounced to allow for clear treatment group separation.

A heatmap of all NMR features, based on HCA, did not result in group clustering by NCAA treatment type, rather sample types were intermingled showing no significant changes between groups (Figure 2B). The NMR metabolomics results are consistent with the MS spectral data. Only the 50 μM HPG samples separated as a unique cluster, similar to what is observed in the heatmap of the LC-MS spectral features (Figure 2A). Taken together, our results suggest that overall, the variability between biological replicates is comparable in magnitude to potential metabolic changes arising from the addition of NCAA in the *E. coli* cell cultures.

This combined MS and NMR metabolomics analysis of NCAA-treated *E. coli* cell cultures demonstrated that these analytical platforms can detect subtle changes in the metabolomes of *E. coli* grown under different culturing conditions, but that additional studies were needed to parse out how these small but potentially significant metabolic changes may impact cellular phenotypes.

### *E. coli* Grown With Non-canonical Amino Acid Under Heat Stress

While planning additional experiments, we evaluated how NCAA are typically used in field work and “real-world” research applications to evaluate how an organism regulates its translational activity in response to environmental (Samo et al., 2014; Hatzenpichler et al., 2016; Leizeaga et al., 2017; Sebastián et al., 2019) or (co)cultivation conditions (Mahdavi et al., 2014; Babin S. A. et al., 2016; Bagert et al., 2016). For such purposes, cellular organisms are often grown for short periods of time under environmental perturbations or cellular stress. We concluded that a more real-world evaluation of a NCAA treatment would include an environmental stressor. Because of the extensive literature available on heat stress response in *E. coli* (Jozefczuk et al., 2010; Ye et al., 2012), we chose temperature increase as an appropriate stressor. Assessing metabolome changes under BONCAT treatment during heat stress would thus not only help clarify how *E. coli* cells are adapting to the incorporation of NCAA, it would also recreate a stress condition that may best reflect “real world” research applications. This rationale thus led to a second set of metabolomics investigations, which utilized high temperature as a stress condition during *E. coli* cell growth with or without a NCAA or MET present.

The heat-treated experiment of *E. coli* consisted of four groups grown at 42°C. Based on the first set of experiments, we narrowed the experimental conditions to 50 μM AHA, HPG or MET. A MET-supplemented culture was again used as a baseline comparison for BONCAT addition, while the control samples contained minimal media. Metabolomics studies and resulting multivariate statistical analysis of LC-MS and NMR metabolite profiles were conducted on heat stressed *E. coli* cell cultures, using the same approach described above for the initial study. As with our initial NCAA addition experiments, physiological data was recorded throughout the growth of the *E. coli* to monitor phenotypic changes and to assess microbial health. Optical density measurements, averaged over biological replicates, were recorded throughout the *E. coli* incubation and growth periods, and indicated that all of the cultures were within 8% OD_600_ of each other, with an average OD_600_ of 1.3 after 210 min of cell growth. Bradford protein assays were utilized to assess protein content and translational activity, prior to intracellular metabolite extraction, and indicated an average protein concentration of 2.1 mg/mL with all samples within 15% of the average concentration (Supplementary Table S2). The range in protein concentration revealed that *E. coli* grown in the presence of AHA, HPG or MET resulted in greater intracellular amounts of proteins than the control cell cultures.

### MS Metabolomics of Cultures Grown With Non-canonical Amino Acids Under Heat Stress

**FIGURE 3:**
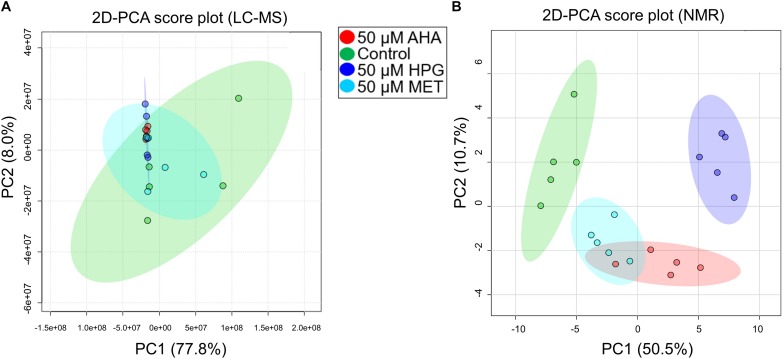
2D PCA plot of MS features and NMR features of heat stressed *E. coli* cultures. **(A)** MS data and **(B)** NMR data are shown. **(A)** The variation within the control group in the MS data completely encompasses the spread of the other sample types. **(B)** Experimental groups show partial separation by NMR. Data is similar to the MS non-stressed PCA plot in that *E. coli* cells with HPG have the greatest separation.

NMR and MS analyses of the intracellular metabolomes of *E. coli* cell cultures grown under heat stress were undertaken utilizing the same analytical approaches described for our first set of experiments. LC-MS analysis identified 5,960 features across all samples. To assess variation and replication trends in the data, PCA analysis was undertaken using the MS metabolite profile data recorded on the heat stressed *E. coli* cell cultures and grown in the presence of AHA, HPG, MET or the no addition control conditions. Resulting 2D-PCA plots did not reveal significant separations between these different groups (Figure 3A), with PC1 accounting for 77.8% of the variance between AHA, HPG or MET treated groups (red, blue, and cyan circles) compared to control (green circle). Principal component 2 accounted for an additional 8% of the variance, reinforcing that similarities rather than differences in metabolic profiles between the treatment groups were most prominent. Variability between the control and the MET-treated cell cultures was as great as the difference between these two groups and the AHA and HPG treated groups. The MET, AHA, and HPG groups clustered more tightly, as illustrated by the shaded 95% confidence intervals of the different groups in the 2D PCA scores plot shown in Figure 3A, compared to that of the control group. The NCAA-treated samples clustered with each other, as did the control and MET-treated *E. coli* samples. This trend was present in the initial set of experiments conducted without heat stress and became more apparent in the PCA analysis of the stressed *E. coli* sample groups (Figure 3A).

An analysis of variance (ANOVA) was also undertaken for the MS-based metabolite profiles of the heat stressed and amino acid treated *E. coli* cell cultures (Supplementary Figure S2). A comparison of the treatment groups to each other, resulted in an *F* value (St Hle and Wold, 1989) of 0.274, and an *F* critical value of 2.61, indicating that the means of the metabolite profiles, i.e., means of the intensities of the MS spectral features, for all the sample groups were not significantly different, and no treatment group differed significantly from the others (Supplementary Table S5). A *post hoc* analysis using Tukey’s honestly significant difference test (Tukey’s HSD test) was conducted with MetaboAnalyst on individual MS spectral features to identify which features accounted most significantly for group differences between the different *E. coli* growth conditions (Supplementary Table S6). The analysis resulted in the identification of 907 features that changed in abundance, Supplementary Figure S2. This is less than 15% of all observed mass features. Each significantly changed feature was subjected to the *post hoc* Tukey HSD test. Features that fell outside the means of other treatment groups is listed (Supplementary Table S6).

A second method employed to assess the data and the impact of AHA and HPG on the intracellular metabolome of *E. coli* was to analyze differentially regulated mass features in the heat stressed and AHA or HPG cell cultures compared to the *E. coli* heat stressed control groups and MET-doped cell cultures. Pairwise comparisons of AHA or HPG treated groups against the MET-treated *E. coli* cell cultures were conducted, as well as comparisons of control group and MET-treated cultures, which found that only 7% of the mass features were significantly different (fold change > 2, *p* < 0.10). Using the same criteria, the AHA and HPG samples were found to contain differentially expressed features at levels of 8 and 19% respectively compared to the MET-doped cultures. These analyses indicate that, while *E. coli* adapts metabolically to the presence of NCAA in its growth medium in the presence of heat stress, each NCAA supplementation impacts the intracellular metabolomes of the *E. coli* cultures in different ways. It also appears that HPG has a greater impact on the metabolome of *E. coli* than AHA, based on pairwise *t*-tests.

**FIGURE 4:**
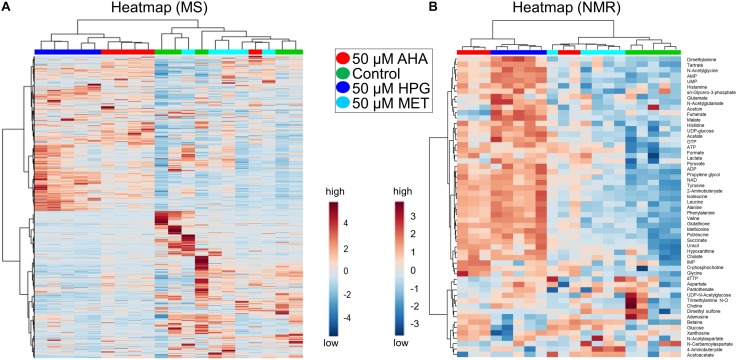
Heatmaps of heat stressed *E. coli* cultures. Data from MS and NMR are shown (**A,B**, respectively). Heat map is coded with blue as low and red as high abundance. Fold change is indicated on the scale. **(A)** AHA and HPG doped stressed *E. coli* cultures show segregated clustering in the MS heatmap of all features (5,960 detected features). **(B)** The NMR heatmap (55 identified features) shows distinct clustering of Control, HPG, and Met, the exception being AHA which had moderate clustering with MET. For enlarged images that show metabolite names and sample identifications see Supplementary Figure S5.

HCA results were plotted on a heatmap to visualize changes in the patterns of individual MS features identified between the NCAA supplemented heat stressed *E. coli* cell cultures. When taking into account all of the MS features, the AHA and HPG supplemented *E. coli* samples separated to a greater extent from the control and MET-supplemented samples, compared to the same groups analyzed in our initial study in the absence of heat stress (Figures 2A, 4A). Although the boundaries between groups were clearer, the HCA did not separate all replicates of a group into unique clusters, nor did the heatmap reveal the presence of a large number of features with significant fold changes (Figure 4A). A heatmap of the top 250 differentiated MS features, as assessed by Tukey’s HSD test, segregated into distinct sample groups best described by growth condition. The heatmap contained blocks of upregulated features that were characteristic of each of the treatment group (Supplementary Figure S3). In this HCA analysis, the AHA and HPG-supplemented groups clustered next to each other while the MET-supplemented and *E. coli* control groups were more similar. The differences between the AHA and HPG treated *E. coli* cell cultures compared to the control and MET-treated groups again showed that MS metabolomics can easily distinguish between the different growth conditions, even when the differentiated features amount to a relatively small proportion of the intracellular metabolome mass spectral features. The color changes on the heatmap indicate fold change, and although not large does reveal that a metabolic adaptation takes place upon addition of the NCAA to the growth medium. To complement the MS-based metabolomics analysis, NMR was utilized to expand metabolite identification and coverage, and to help with the assessment of the potential biological impact of those metabolic adaptations on the cellular phenotypes of *E. coli.*

### NMR Metabolomics Analysis of Cultures Grown With Non-canonical Amino Acids Under Heat Stress

From analysis of 1D ^1^H NMR spectra and spectral profiling using the Chenomx software, 55 metabolites were identified and quantified from intracellular metabolite extracts of the heat-stressed, NCAA-supplemented *E. coli* cultures (Supplementary Table S7). While the MS metabolomics data demonstrated the presence of a certain degree of metabolic adaptation occurring in these cell cultures, the 55 metabolites annotated and validated by NMR provided some clues as to which metabolic pathways may be involved in these metabolic adaptations. The NMR metabolomics studies of the heat stressed, AHA, HPG, and MET supplemented *E. coli* cell cultures employed the same experimental workflow used for examining the intracellular metabolomes of the *E. coli* cell cultures in absence of heat stress.

Group separations were assessed using PCA. The resulting 2D-PCA scores plots (Figure 3B) revealed that HPG, AHA, and MET-supplemented *E. coli* groups could be separated based on their distinct NMR-based metabolome profiles from the control group. Furthermore, the AHA and HPG-treated groups also separated from each other based on distinct metabolite patterns (Figure 3B, red and purple 95% confidence interval circles), while the metabolic profile of the MET supplemented group overlapped with that of the AHA-treated group (Figure 3B, red and cyan 95% confidence intervals). Analysis of loading factors (Supplementary Table S8) contributing to PC1 and PC2 of the 2D PCA-score plots revealed that betaine, xanthosine, *N*-carbamoyl-aspartate, glucose, 4-aminobutyrate, adenosine contributed significantly to PC1, which accounted for 50.5% of the variance, while PC2 accounted for an additional 10.7%.

Although the samples separated from each other primarily along the PC1 axis, as with the MS metabolomics findings, the variation between sample replicates was rather large and resulted in minimal separation by treatment type. In other words, although metabolic adaptations occur within the cell in the presence of NCAA under heat stress, those metabolic responses appear to be rather limited and do not seem to suggest that a significant overhaul of the metabolic machinery of *E. coli* is taking place.

HCA, schematically represented as a heatmap of relative NMR metabolite abundance, was employed to further evaluate differences among the metabolite profiles of each *E. coli* treatment group (Figure 4B). This heatmap was generated from changes in metabolite concentrations observed for the 54 metabolites that were identified by NMR. The control and MET-supplemented groups clustered more closely, while the NCAA treated groups formed a second cluster. The one exception was a replicate from the *E. coli* cell cultures supplemented with MET (Figure 4B). Consistent with the HCA analysis of the MS spectral features, the heatmap representation of the NMR-based metabolite profiles suggest that although AHA or HPG supplementation does induce changes in intracellular metabolome of *E. coli*, no dramatic metabolic alterations appeared to have taken place within the cells.

### Combined Pathway Analysis of Heat Stressed *E. coli* Cultures

**FIGURE 5:**
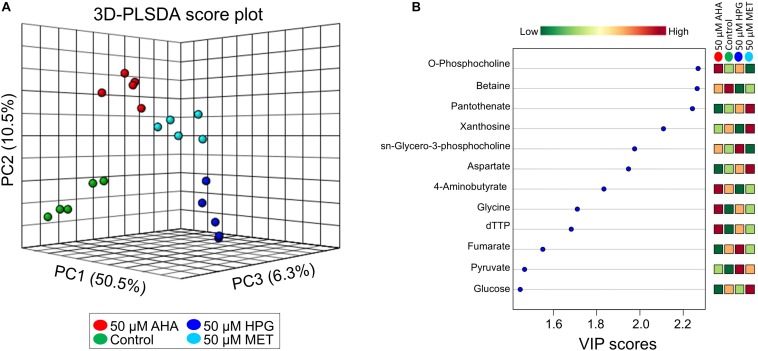
3D-PLSDA of metabolites as identified by NMR from the heat stressed non-canonical amino acid doped *E. coli* cultures and corresponding VIP scores table. **(A)** The PLSDA shows distinct separation between the doping groups **(B)** VIP shows the top 12 metabolites (out of 55 total metabolites) that contributed the most to the variation between sample type**s.**

The NMR metabolomics data provided important information about potential changes in metabolic pathway usage based on a metabolic pathway impact analysis that was conducted using MetaboAnalyst. Partial least squares discriminate analysis (PLS-DA) (Figure 5A), with resulting variable importance in projection (VIP) scores for metabolites that have the highest discriminatory power among the treatment groups was used (Cho et al., 2008). Metabolites that contributed most to the separation of the different sample groups are listed in the VIP scores plot and revealed interesting trends between the different cell culture treatment groups (Figure 5B). This analysis indicated that NCAA addition impacted amino acid, protein, and lipid metabolism. Intermediates in central carbon metabolism via lipid and amino acid synthesis and TCA cycle related metabolites, including aspartate, glycine, fumarate, glucose, pyruvate, malate, and 4-aminobutyrate were altered as a result of AHA or HPG supplementation in the growth medium, resulting in high differentiation between sample treatments for these molecules (Supplementary Tables S9, S10). Several metabolites related to pyruvate metabolism demonstrated a statistically significant differentiation between the NCAA-treated *E. coli* groups, supporting the idea that TCA cycle activity was altered in the AHA and HPG supplemented *E. coli* cell cultures. Intracellular levels of pyruvate, succinate, formate, and acetate were found to be higher in the *E. coli* cell cultures grown under heat stress and supplemented with NCAA. Metabolites associated with purine and amino acid metabolism, like xanthosine, dTTP, glycine and adenosine, also pointed to metabolic networks related to energy production as being altered in the NCAA treated cells. In addition to amino acid biosynthesis, glycerophospholipid metabolism was dysregulated as a result of NCAA incorporation with *O*-phosphocholine, and sn-glycero-3-phosphocholine are present at higher concentrations in the HPG and AHA supplemented cultures. Metabolites associated with lipid, amino acid and purine metabolism were consistently higher in abundance in the NCAA-treated *E. coli* groups then the control and methionine treated *E. coli*.

While NCAA addition impacted TCA cycle activity within the cell and potentially energy production via amino acid, purine and lipid metabolism, the implications of such metabolic changes remain unclear. Amino acid biosynthesis and degradation were altered, as leucine, MET, and tyrosine were present at higher concentrations in the AHA and HPG doped samples than in the control and the MET doped samples (Supplementary Figure S4). These results indicate that amino acid metabolism in *E. coli* is altered upon addition of a NCAA suggesting that leucine, MET, and tyrosine catabolism may be suppressed in the AHA and HPG-supplemented cell cultures, or that other metabolites serve as metabolic precursors for energy production under these conditions, sparing the utilization of leucine, MET, and tyrosine for such purpose.

In the heat exposed, AHA or HPG doped growth conditions, intracellular levels of amino acids were found to be higher than in control or MET-treated groups (Supplementary Tables S9, S10), including higher abundance of acetylated amino acids like *N*-actylglycine, *N*-acetylgultamate, and *N*-acetylaspartate. Acetylated amino acids could represent breakdown products of proteins that have been acetylated (Arnesen, 2011) and have been reported to be used for metabolic adaptations of microorganisms. Protein acetylation is a common post- and co-translational modification process for metabolic enzymes involved in central metabolism (Christensen et al., 2019). This modification is usually found on the side chains of amino acids, not on protein backbone residues, and could explain why free acetylated amino acids were detected in high abundances in the AHA and HPG-treated *E. coli* cell cultures. Amino acid acetylation could also be indicative of a higher rate of post translational modifications in the NCAA doped samples (Elf and Ehrenberg, 2005), which would suggest changes in the accuracy of protein translation, protein signaling, and protein-protein interactions. There are differing hypotheses on the implications of acetylation on protein degradation. One school of thought indicates that it is protective (Carabetta and Cristea, 2017), while more recent studies have reported the opposite (Arnesen, 2011). Additional studies are needed to fully elucidate the impact of AHA or HPG supplementation on protein acetylation.

### Screening for Potential Degradation Products of AHA and HPG

A question remaining to be address on the use of BONCAT relates to whether protein synthesis indeed serves as the only sink for the incorporation of NCAA or whether some organisms could be capable of metabolizing NCAA for their energetic needs. In an attempt to provide answers to this issue, our LC-MS data was searched for potential breakdown and conversion products of AHA and HPG as predicted from KEGG pathways and assuming that AHA or HPG could serve as substrates for enzymatic conversions. Potential compounds of interest included *N*-formyl-AHA and *N*-formyl-HPG as well as AHA/HPG versions of 4-(methylsulfanyl)-2-oxobutanoate. Other degradation products were ruled out because they all required activation of the MET-sulfur functional group, which is absent from both HPG and AHA. No features that matched these suspected products were detected. This implies that breakdown of AHA and HPG is not a major metabolic activity of *E. coli*, and that the main sink for AHA and HPG is, indeed, protein synthesis.

### Summary

Utilizing NMR and LC-MS approaches, we were able to establish that NCAA addition can cause metabolic perturbation and adaptation in *E. coli*, especially when the bacteria are subjected to heat stress. MS analyses indicated that the presence of NCAA altered the concentration of approximately 15% of the global mass features identified based on ANOVA. To put this into perspective, the addition of MET altered the abundance of 7% of the all the mass spectral features detected in the *E. coli* cells. This mild perturbation is consistent with previous studies that have investigated the impact of AHA or HPG replacement of MET (Dieterich et al., 2006; Bagert et al., 2014; Hatzenpichler et al., 2014, 2016; Hatzenpichler and Orphan, 2015; Landgraf et al., 2015; Calve et al., 2016; Lehner et al., 2017). Although the observed metabolic changes were mild, the heatmaps and 2D-PCA score plots highlighted trends between the different *E. coli* treatment groups. HCA also showed that while AHA and HPG addition impacts the global metabolism of *E. coli* to some extent, the lack of group separation based on distinct metabolite profiles suggests that these metabolic changes are minimal under regular growth conditions and become more pronounced when cells are subjected to heat stress.

The global NMR and MS data are consistent in revealing the absence of significant group separation between the different *E. coli* cell cultures. The largest difference between groups was observed for the HPG-treated cells. Along this same trend, the AHA- and MET-doped cultures were more similar in metabolite profiles, group clustering, and metabolic change at the individual metabolite level. HPG seemed to perturb *E. coli* to a larger extent than AHA based on paired *t*-tests which had 19 and 8% of metabolites changing, respectively. This was not expected because the differential impact of AHA and HPG on *E. coli* had not been reported previously.

The NMR data lent power to our analysis in the form of metabolite annotation and validation. Changes in specific metabolite levels indicated that pyruvate metabolism and intermediates of the TCA cycle were affected. Changes in central carbon metabolism is a common stress response in *E. coli*, so the perturbations we observed as a result of NCAA additions are consistent with this archetypical stress response (Jozefczuk et al., 2010). Along with TCA metabolites, glycerophospholipids, amino acids and acetylated amino acids were detected at higher concentrations in the AHA and HPG supplemented *E. coli* samples.

In-depth NMR and MS metabolomic analyses show that supplementing *E. coli* cultures with NCAA has an impact on the concentration of specific metabolites leading to a metabolic adjustment. This should serve as a cautionary note to scientists about how and when NCAA can be used. Our data implies that the common practices of using optical density for cells in culture or behavioral analyses for multicellular species to assess the impact of NCAA supplementation are not telling a complete story. Metabolic profiles do change, but our overall assessment is that under normal or even moderately stressful growth conditions, NCAA doping causes minor perturbations to the overall metabolic homeostasis of microbial cells.

## Data Availability Statement

The datasets generated for this study can be found in the Metabolomics Work Bench, https://www.metabolomicsworkbench.org/data/MWTABMetadata4.php?F=kfsteward_20191206_111451_mwtab_analysis_1.txt&Mode=Study&DataMode=AllData&StudyType=MS#DataTabs.

## Author Contributions

RH, BB, VC, and KS conceptualized and designed the study. RH, BS, MD, BE, and NB worked on the experimental setup and manipulations. All authors analyzed and interpreted the data, critically revised the manuscript for important intellectual content. KS, BB, RH, and VC drafted the manuscript.

## Conflict of Interest

The authors declare that the research was conducted in the absence of any commercial or financial relationships that could be construed as a potential conflict of interest.

## Footnotes

1 https://www.bruker.com/service/support-upgrades/software-downloads/nmr.html

2 https://www.chenomx.com/products/

3 https://www.chenomx.com/products/

4 https://www.bruker.com/service/support-upgrades/software-downloads/nmr.html
