# Supplementary material for: Metabolic Implications of Using BioOrthogonal Non-Canonical Amino Acid Tagging (BONCAT) for Tracking Protein Synthesis
Source: Front Microbiol. 2020 Feb 13;11:197. doi: 10.3389/fmicb.2020.00197 (PMC7031258; doi:10.3389/fmicb.2020.00197)
Supplement: Supplementary file 1 [file Data_Sheet_1.pdf]

**Table S1**. OD growth curves. All within 8.0% of the overall average at 1.3.			
**Sample**	**OD**		**Average OD**
	**0 min**	**210 min**	
C1	0.871	1.231	1.217
C2	0.871	1.264	
C3	0.871	1.224	
C4	0.871	1.180	
C5	0.871	1.184	
A1	0.870	1.290	1.335
A2	0.870	1.349	
A3	0.870	1.412	
A4	0.870	1.276	
A5	0.870	1.347	
H1	0.872	1.493	1.418
H2	0.872	1.311	
H3	0.872	1.354	
H4	0.872	1.456	
H5	0.872	1.474	
M1	0.871	1.283	1.265
M2	0.871	1.230	
M3	0.871	1.258	
M4	0.871	1.334	
M5	0.871	1.222	

Table S2. Bradford assay protein concentrations. All within 15.0% of the overall average at 2.1.

Sample #	mg/mL	Average mg/mL
1	1.94	1.83
2	1.90	
3	1.87	
4	1.74	
5	1.69	
6	1.91	1.94
7	1.91	
8	1.91	
9	2.16	
10	1.82	
11	2.15	2.36
12	2.37	
13	2.45	
14	2.25	
15	2.56	
16	2.37	2.43
17	2.35	
18	2.40	
19	2.40	
20	2.63	

Table S3. ANOVA single factor MS data in initial study

SUMMARY						
**Groups**	**Count**	**Sum**	**Average**	**Variance**		
CTRL avg	4036	9.57E+08	237029	5.66E+11		
M1 avg	4036	9.99E+08	247604	7.78E+11		
M50 avg	4036	9.43E+08	233555	6.93E+11		
A1 avg	4036	9.11E+08	225683	6.16E+11		
A50 avg	4036	8.59E+08	212862	5.88E+11		
H1 avg	4036	9.24E+08	228978	1.44E+12		
H50 avg	4036	8.56E+08	212201	7.09E+11		
**ANOVA**						
**Source of Variation**	**SS**	**df**	**MS**	**F**	**P-value**	**F crit**
Between Groups	3.96E+12	6	6.60E+11	0.85787	0.52509	2.09892
Within Groups	2.17E+16	28245	7.69E+11			
Total	2.17E+16	28251				

ANOVA test on non-stressed data show that all are the same, because *p*-value is greater than the alpha value at 0.05, and the F value is much smaller than the F critical value.

Table S4. NMR metabolites in initial study.

Metabolite	Control		Met-1mM		Met-50µM		AHA-1mM		AHA-50µM		HPG-1mM		HPG-50µM	
	Average	SD	Average	SD	Average	SD	Average	SD	Average	SD	Average	SD	Average	SD
2-Aminobutyrate	3.0	0.3	1.8	0.5	3.3	0.7	2.7	1.8	2.5	0.8	1.0	0.4	1.2	0.0
4-Aminobutyrate	159.7	44.6	138.3	63.4	143.8	74.5	138.3	53.8	106.3	15.1	198.6	45.0	130.2	37.1
Acetate	613.4	225.4	747.9	245.0	691.1	134.6	675.0	255.0	429.4	85.3	604.8	184.3	345.5	105.8
Acetoin	2.6	0.5	3.0	1.0	2.1	0.9	2.2	1.7	1.8	0.4	1.0	0.6	1.4	0.5
Adenosine	1.6	1.1	2.3	1.9	1.3	0.9	1.3	0.7	1.6	0.9	1.1	1.2	0.0	0.0
Alanine	123.6	28.8	119.1	19.4	125.7	6.2	85.3	43.9	71.0	10.9	65.7	43.2	57.7	7.4
AMP	10.3	4.4	6.6	6.6	11.9	2.7	6.4	2.2	3.4	1.9	5.8	1.2	8.3	0.9
Aspartate	5.0	0.8	4.3	1.5	4.7	1.7	9.6	10.7	3.5	0.6	10.2	6.8	3.6	0.8
Dimethylamine	1.9	0.3	2.0	0.3	1.8	0.2	1.5	0.5	1.0	0.2	1.1	0.2	1.4	0.3
dTTP	28.4	6.2	30.9	7.6	34.6	6.6	23.2	5.0	18.2	2.5	21.5	3.9	11.4	1.5
Formate	274.8	86.8	369.7	24.8	470.7	136.5	295.6	62.4	249.4	46.8	286.9	88.0	170.7	20.1
Fumarate	5.6	2.0	4.2	0.3	3.5	0.7	5.7	2.8	4.5	0.5	3.1	0.6	5.6	1.0
Glucose	288.5	243.0	250.0	196.8	348.6	257.8	167.9	201.8	281.0	246.7	49.5	110.7	445.6	88.4
Glucose-1-phosphate	6.2	1.3	7.0	1.4	7.1	1.3	4.9	1.3	3.8	0.8	3.9	1.1	5.7	0.9
Glutamate	17.1	2.2	66.1	70.9	58.7	70.0	20.3	7.9	18.6	5.8	13.0	2.6	15.7	5.7
Glutathione	36.1	4.6	47.8	7.5	54.4	21.2	28.4	11.3	21.0	4.7	32.6	6.6	23.7	2.4
Glycine	15.3	3.7	21.4	3.6	15.9	7.0	13.0	2.9	14.6	2.7	35.5	11.1	10.4	1.1
Histidine	1.1	0.5	1.5	0.7	1.1	0.3	1.3	0.5	1.2	0.4	1.0	0.4	0.8	0.1
Hypoxanthine	9.3	3.1	12.2	1.7	11.2	3.7	5.5	1.6	4.7	1.1	6.5	2.2	3.4	0.5
Isoleucine	2.6	0.5	2.5	0.3	3.6	2.0	4.0	1.8	1.8	0.3	2.3	0.5	1.1	0.2
Lactate	118.1	62.0	210.3	38.9	158.5	61.8	73.9	31.2	46.5	13.8	71.5	65.7	37.2	12.2
Leucine	4.0	0.4	4.3	0.6	4.2	0.4	5.3	1.7	3.6	0.3	3.3	0.6	3.0	0.5
Malate	59.8	9.3	55.4	11.5	52.3	4.7	72.2	35.4	47.1	6.0	50.1	8.5	56.6	8.9
Methionine	3.2	1.0	361.1	34.5	16.9	2.9	5.9	2.6	2.7	0.4	0.0	0.0	0.0	0.0
N-Acetylaspartate	15.5	3.4	20.2	16.1	11.8	2.8	21.0	17.2	17.9	8.1	12.9	14.1	16.8	3.5
N-Acetylglycine	1.1	0.2	2.1	0.5	1.7	0.3	1.0	0.4	0.7	0.2	0.7	0.2	0.5	0.0
NAD^+	22.2	5.0	24.1	4.9	27.5	3.9	20.8	7.6	14.3	1.4	21.4	5.4	10.3	1.5
$NADP^+$	3.4	0.8	3.5	0.5	3.6	1.0	2.2	1.0	2.0	0.7	1.9	0.9	1.6	0.4
Pantothenate	1.2	0.3	1.1	0.3	1.6	0.6	1.2	0.5	0.8	0.1	1.4	0.5	0.4	0.1
Phenylalanine	2.5	0.6	2.2	1.0	2.2	1.7	2.0	0.4	1.7	0.5	2.1	0.7	1.7	0.3
Propylene glycol	1.7	0.4	1.9	0.5	1.7	0.3	1.6	0.7	1.5	0.5	1.2	0.5	1.1	0.2
Putrescine	53.5	18.5	64.6	30.8	52.2	13.7	50.8	21.4	49.7	13.7	59.3	25.7	36.0	9.7
Pyruvate	95.0	28.0	129.4	42.8	105.9	59.5	54.8	35.7	34.7	23.8	20.3	19.5	47.3	10.5
Succinate	97.3	27.2	119.1	8.7	143.4	57.3	92.8	32.3	67.7	27.9	114.9	30.4	39.9	5.7
Tyrosine	9.6	2.5	10.4	1.2	10.4	3.9	7.7	3.0	5.4	1.1	5.9	2.7	7.1	1.5
UDP-glucose	15.9	4.6	22.3	4.7	16.9	3.1	15.0	7.6	10.7	3.1	9.4	3.9	9.1	1.0
UDP-N-Acetylglucosamine	3.7	0.6	3.9	0.8	4.2	1.8	2.5	0.9	2.4	0.3	0.8	1.8	3.6	0.4
UMP	10.4	1.3	10.8	2.2	9.8	2.3	8.4	4.3	5.7	2.0	4.4	3.9	7.5	1.1
Uracil	11.6	3.2	10.9	5.1	13.5	2.2	6.7	2.7	7.7	1.9	8.1	4.4	3.1	0.4
Valine	14.3	2.6	17.8	4.6	16.1	2.4	13.0	5.2	9.7	2.2	8.0	2.2	6.4	0.6

*Assignment with best-matched signals, all others validated.

Table S5. ANOVA single factor MS heat-stressed data.

SUMMARY						
**Groups**	**Count**	**Sum**	**Average**	**Variance**		
CTRL avg	5960	9.80E+08	164503	1.53E+12		
MET avg	5960	1.10E+09	184611	2.22E+12		
AHA avg	5960	9.93E+08	166573	1.65E+12		
HPG avg	5960	1.01E+09	169402	1.83E+12		
**ANOVA**						
**Source of Variation**	**SS**	**df**	**MS**	**F**	**P-value**	**F crit**
Between Groups	1.49E+12	3	4.95E+11	0.27394	0.84424	2.60528
Within Groups	4.31E+16	23836	1.81E+12			
Total	4.31E+16	23839				

Table S6. Tukey's results from MS ANOVA heat-stressed analysis. FC and t-test at 0.1 M v. C = 438 features, M v. H = 1119 features, M v. A = 465 features.

MZ feature	f.value	p.value	RT	FDR	Tukey's HSD
128.0671	169.6	2.46E-12	11.609	8.79E-09	HPG-AHA; HPG-CTRL; MET-HPG
334.9927	148.12	7.00E-12	11.155	1.25E-08	HPG-AHA; HPG-CTRL; MET-HPG
82.06306	109.35	7.15E-11	10.146	8.52E-08	HPG-AHA; HPG-CTRL; MET-HPG
614.1522	86.512	4.20E-10	9.3764	2.90E-07	CTRL-AHA; HPG-AHA; HPG-CTRL; MET-HPG
613.1495	85.707	4.51E-10	9.346	2.90E-07	CTRL-AHA; HPG-AHA; MET-AHA; HPG-CTRL; MET-HPG
615.1487	84.844	4.86E-10	9.313	2.90E-07	CTRL-AHA; HPG-AHA; HPG-CTRL; MET-HPG
635.1304	82.439	6.03E-10	9.2194	3.04E-07	CTRL-AHA; HPG-AHA; HPG-CTRL; MET-HPG
219.076	81.135	6.80E-10	9.1676	3.04E-07	CTRL-AHA; HPG-AHA; HPG-CTRL; MET-CTRL; MET-HPG
997.2834	77.759	9.34E-10	9.0297	3.71E-07	CTRL-AHA; HPG-AHA; MET-AHA; HPG-CTRL; MET-HPG
999.2831	75.321	1.18E-09	8.9267	4.19E-07	CTRL-AHA; HPG-AHA; MET-AHA; HPG-CTRL; MET-HPG
425.0357	74.471	1.29E-09	8.89	4.19E-07	CTRL-AHA; MET-AHA; HPG-CTRL; MET-CTRL; MET-HPG
613.1494	71.637	1.72E-09	8.7648	4.63E-07	CTRL-AHA; HPG-AHA; MET-AHA; HPG-CTRL; MET-CTRL; MET-HPG
651.1023	71.206	1.80E-09	8.7454	4.63E-07	HPG-AHA; HPG-CTRL; MET-HPG
356.986	71.127	1.81E-09	8.7419	4.63E-07	HPG-AHA; HPG-CTRL; MET-HPG
84.95764	70.038	2.03E-09	8.6922	4.84E-07	CTRL-AHA; HPG-AHA; MET-AHA; HPG-CTRL; MET-CTRL; MET-HPG
255.9729	68.546	2.38E-09	8.6231	5.32E-07	CTRL-AHA; HPG-AHA; MET-AHA; HPG-CTRL; MET-CTRL; MET-HPG
622.0188	63.458	4.20E-09	8.3762	8.84E-07	CTRL-AHA; MET-AHA; HPG-CTRL; MET-CTRL; MET-HPG
159.1455	60.438	6.01E-09	8.221	1.09E-06	CTRL-AHA; MET-AHA; HPG-CTRL; MET-HPG
614.1521	60.405	6.04E-09	8.2193	1.09E-06	CTRL-AHA; HPG-AHA; HPG-CTRL; MET-CTRL; MET-HPG
140.0778	60.285	6.12E-09	8.2129	1.09E-06	CTRL-AHA; MET-AHA; HPG-CTRL; MET-CTRL; MET-HPG
823.4129	58.394	7.73E-09	8.1119	1.32E-06	HPG-AHA; HPG-CTRL; MET-HPG
173.1247	57.464	8.69E-09	8.0611	1.41E-06	CTRL-AHA; HPG-AHA; MET-AHA
168.072	56.271	1.01E-08	7.9947	1.57E-06	CTRL-AHA; HPG-AHA; MET-AHA; HPG-CTRL; MET-HPG
921.9953	54.912	1.21E-08	7.9176	1.80E-06	CTRL-AHA; MET-AHA; HPG-CTRL; MET-CTRL; MET-HPG
125.9832	54.396	1.29E-08	7.8879	1.85E-06	CTRL-AHA; HPG-AHA; MET-AHA; HPG-CTRL; MET-CTRL; MET-HPG
384.9744	54.105	1.35E-08	7.8711	1.85E-06	CTRL-AHA; HPG-AHA; MET-AHA; HPG-CTRL; MET-CTRL; MET-HPG
824.416	52.933	1.58E-08	7.8023	2.09E-06	CTRL-AHA; HPG-AHA; HPG-CTRL; MET-HPG
939.3285	52.321	1.71E-08	7.7658	2.19E-06	CTRL-AHA; HPG-AHA; HPG-CTRL; MET-HPG
767.4632	51.419	1.94E-08	7.7113	2.40E-06	CTRL-AHA; HPG-AHA; HPG-CTRL; MET-HPG
881.3702	50.845	2.11E-08	7.6762	2.45E-06	CTRL-AHA; HPG-AHA; HPG-CTRL; MET-HPG
317.1124	50.803	2.12E-08	7.6736	2.45E-06	CTRL-AHA; HPG-AHA; MET-AHA
663.4688	50.168	2.32E-08	7.6343	2.59E-06	HPG-AHA; HPG-CTRL; MET-HPG
261.9587	49.961	2.39E-08	7.6214	2.59E-06	CTRL-AHA; HPG-AHA; MET-AHA; HPG-CTRL; MET-HPG
300.2842	49.76	2.46E-08	7.6088	2.59E-06	CTRL-AHA; MET-AHA; HPG-CTRL; MET-CTRL; MET-HPG
134.0423	48.977	2.76E-08	7.5594	2.82E-06	CTRL-AHA; HPG-AHA; MET-AHA; HPG-CTRL; MET-HPG
283.2581	48.025	3.17E-08	7.4983	3.15E-06	CTRL-AHA; MET-AHA; HPG-CTRL; MET-CTRL; MET-HPG
384.1085	47.391	3.49E-08	7.457	3.31E-06	HPG-AHA; MET-AHA; HPG-CTRL; MET-HPG
765.4545	47.336	3.52E-08	7.4534	3.31E-06	HPG-AHA; HPG-CTRL; MET-HPG
158.0435	46.817	3.81E-08	7.4192	3.42E-06	CTRL-AHA; HPG-CTRL; MET-CTRL
118.1198	46.785	3.83E-08	7.4171	3.42E-06	CTRL-AHA; HPG-AHA; MET-AHA; HPG-CTRL; MET-HPG
825.4134	46.165	4.21E-08	7.3758	3.48E-06	HPG-AHA; HPG-CTRL; MET-HPG
941.3278	46.164	4.21E-08	7.3757	3.48E-06	CTRL-AHA; HPG-AHA; HPG-CTRL; MET-HPG
690.4968	46.136	4.23E-08	7.3739	3.48E-06	HPG-AHA; HPG-CTRL; MET-HPG
882.3721	46.048	4.29E-08	7.3679	3.48E-06	CTRL-AHA; HPG-AHA; HPG-CTRL; MET-HPG
226.1151	45.591	4.60E-08	7.3371	3.66E-06	HPG-AHA; HPG-CTRL; MET-HPG
132.0004	44.09	5.84E-08	7.2338	4.46E-06	CTRL-AHA; HPG-AHA; MET-AHA; HPG-CTRL; MET-HPG
883.3709	44.063	5.86E-08	7.2319	4.46E-06	CTRL-AHA; HPG-AHA; HPG-CTRL; MET-HPG
168.0735	43.637	6.28E-08	7.2021	4.62E-06	CTRL-AHA; HPG-AHA; MET-AHA; HPG-CTRL; MET-HPG
622.0184	43.59	6.33E-08	7.1988	4.62E-06	CTRL-AHA; HPG-AHA; HPG-CTRL; MET-CTRL; MET-HPG
59.07153	42.815	7.18E-08	7.1436	5.11E-06	CTRL-AHA; HPG-AHA; HPG-CTRL; MET-CTRL; MET-HPG
395.6704	42.686	7.34E-08	7.1344	5.11E-06	CTRL-AHA; HPG-AHA; MET-AHA; HPG-CTRL; MET-HPG
691.5001	42.608	7.43E-08	7.1288	5.11E-06	HPG-AHA; HPG-CTRL; MET-HPG
693.5094	42.211	7.94E-08	7.1001	5.36E-06	HPG-AHA; HPG-CTRL; MET-HPG
662.4658	41.942	8.31E-08	7.0805	5.46E-06	HPG-AHA; HPG-CTRL; MET-HPG
837.6148	41.878	8.40E-08	7.0758	5.46E-06	CTRL-AHA; HPG-AHA; MET-AHA; HPG-CTRL; MET-HPG
377.1388	41.248	9.34E-08	7.0295	5.92E-06	HPG-AHA; HPG-CTRL; MET-HPG
391.2777	41.184	9.45E-08	7.0247	5.92E-06	CTRL-AHA; HPG-CTRL; MET-CTRL; MET-HPG
470.2531	40.06	1.15E-07	6.9404	7.07E-06	CTRL-AHA; HPG-AHA; HPG-CTRL; MET-HPG
921.9952	39.715	1.22E-07	6.914	7.39E-06	CTRL-AHA; HPG-AHA; HPG-CTRL; MET-CTRL; MET-HPG
178.9888	39.05	1.37E-07	6.8627	8.17E-06	HPG-AHA; HPG-CTRL; MET-HPG
766.4573	38.761	1.44E-07	6.8403	8.47E-06	HPG-AHA; HPG-CTRL; MET-HPG
144.0445	38.441	1.53E-07	6.8151	8.71E-06	CTRL-AHA; HPG-AHA; MET-AHA; HPG-CTRL; MET-CTRL; MET-HPG
202.1014	38.428	1.53E-07	6.8141	8.71E-06	HPG-AHA; HPG-CTRL; MET-HPG
523.1978	38.265	1.58E-07	6.8012	8.83E-06	CTRL-AHA; HPG-CTRL; MET-CTRL
522.1944	38.001	1.66E-07	6.7803	9.01E-06	CTRL-AHA; MET-AHA; HPG-CTRL; MET-CTRL; MET-HPG
692.5048	37.983	1.66E-07	6.7788	9.01E-06	HPG-AHA; HPG-CTRL; MET-HPG
117.1357	37.478	1.83E-07	6.7384	9.74E-06	CTRL-AHA; MET-AHA; HPG-CTRL; MET-HPG
408.0006	37.041	1.98E-07	6.703	1.04E-05	CTRL-AHA; HPG-AHA; HPG-CTRL; MET-HPG
718.0465	36.827	2.06E-07	6.6856	1.07E-05	CTRL-AHA; HPG-AHA; MET-AHA; HPG-CTRL; MET-HPG
614.152	36.646	2.13E-07	6.6708	1.09E-05	CTRL-AHA; HPG-AHA; HPG-CTRL; MET-CTRL; MET-HPG

283.258	36.304	2.28E-07	6.6426	1.15E-05	CTRL-AHA; MET-AHA; HPG-CTRL; MET-CTRL; MET-HPG
688.4808	35.659	2.58E-07	6.5888	1.28E-05	HPG-AHA; HPG-CTRL; MET-HPG
462.0506	35.564	2.63E-07	6.5808	1.29E-05	HPG-AHA; HPG-CTRL; MET-HPG
424.0967	35.002	2.93E-07	6.5331	1.42E-05	HPG-AHA; HPG-CTRL; MET-HPG
219.13	34.528	3.22E-07	6.4924	1.53E-05	CTRL-AHA; HPG-AHA; HPG-CTRL; MET-HPG
122.0933	33.615	3.87E-07	6.4125	1.81E-05	CTRL-AHA; HPG-CTRL; MET-CTRL; MET-HPG
261.1229	33.518	3.95E-07	6.404	1.81E-05	CTRL-AHA; HPG-AHA; MET-AHA; HPG-CTRL; MET-HPG
598.17	33.508	3.95E-07	6.4031	1.81E-05	CTRL-AHA; HPG-AHA; MET-AHA; HPG-CTRL; MET-HPG
605.0016	32.626	4.74E-07	6.324	2.15E-05	CTRL-AHA; HPG-AHA; MET-AHA; HPG-CTRL; MET-HPG
609.6501	32.297	5.08E-07	6.2941	2.25E-05	CTRL-AHA; HPG-AHA; MET-AHA; HPG-CTRL; MET-HPG
303.136	32.251	5.13E-07	6.2898	2.25E-05	CTRL-AHA; MET-AHA; HPG-CTRL; MET-HPG
172.0386	32.225	5.16E-07	6.2874	2.25E-05	CTRL-AHA; MET-AHA; HPG-CTRL; MET-CTRL; MET-HPG
395.6703	32.074	5.33E-07	6.2736	2.29E-05	CTRL-AHA; HPG-AHA; MET-AHA; HPG-CTRL; MET-HPG
608.6577	31.325	6.25E-07	6.2039	2.65E-05	CTRL-AHA; HPG-AHA; MET-AHA; HPG-CTRL; MET-HPG
631.1122	31.244	6.36E-07	6.1963	2.65E-05	HPG-AHA; HPG-CTRL; MET-HPG
535.8933	31.238	6.37E-07	6.1957	2.65E-05	CTRL-AHA; HPG-AHA; HPG-CTRL; MET-HPG
423.9749	30.892	6.87E-07	6.163	2.78E-05	CTRL-AHA; HPG-AHA; HPG-CTRL; MET-HPG
425.0983	30.864	6.91E-07	6.1604	2.78E-05	HPG-AHA; HPG-CTRL; MET-HPG
598.6707	30.855	6.93E-07	6.1595	2.78E-05	CTRL-AHA; HPG-AHA; MET-AHA; HPG-CTRL; MET-HPG
622.0182	30.722	7.13E-07	6.1469	2.80E-05	CTRL-AHA; HPG-AHA; HPG-CTRL; MET-CTRL; MET-HPG
702.0789	30.687	7.19E-07	6.1435	2.80E-05	CTRL-AHA; HPG-AHA; HPG-CTRL; MET-HPG
394.9512	30.673	7.21E-07	6.1421	2.80E-05	HPG-AHA; HPG-CTRL; MET-HPG
597.6689	30.551	7.40E-07	6.1305	2.82E-05	CTRL-AHA; HPG-AHA; MET-AHA; HPG-CTRL; MET-HPG
689.4839	30.513	7.47E-07	6.1268	2.82E-05	HPG-AHA; HPG-CTRL; MET-HPG
437.2066	30.495	7.50E-07	6.1251	2.82E-05	CTRL-AHA; MET-AHA; HPG-CTRL; MET-HPG
718.0466	30.033	8.31E-07	6.0804	3.09E-05	CTRL-AHA; HPG-AHA; HPG-CTRL; MET-HPG
859.2791	29.633	9.09E-07	6.0414	3.35E-05	HPG-AHA; HPG-CTRL; MET-HPG
239.1244	29.511	9.35E-07	6.0293	3.41E-05	MET-AHA; HPG-CTRL; MET-CTRL; MET-HPG
386.0198	29.319	9.76E-07	6.0103	3.53E-05	CTRL-AHA; HPG-AHA; HPG-CTRL; MET-HPG
717.4595	29.207	1.00E-06	5.9992	3.58E-05	HPG-AHA; HPG-CTRL; MET-HPG
425.0079	28.886	1.08E-06	5.967	3.82E-05	CTRL-AHA; MET-AHA; HPG-CTRL; MET-HPG
307.5795	28.804	1.10E-06	5.9588	3.85E-05	HPG-AHA; HPG-CTRL; MET-HPG
146.0365	28.661	1.14E-06	5.9443	3.95E-05	CTRL-AHA; MET-AHA; HPG-CTRL; MET-HPG
445.9557	28.488	1.18E-06	5.9268	4.05E-05	HPG-AHA; HPG-CTRL; MET-HPG
144.0455	28.47	1.19E-06	5.925	4.05E-05	CTRL-AHA; MET-AHA; HPG-CTRL; MET-CTRL; MET-HPG
198.0808	28.36	1.22E-06	5.9137	4.11E-05	CTRL-AHA; MET-AHA; HPG-CTRL; MET-CTRL; MET-HPG
220.1319	28.232	1.26E-06	5.9007	4.20E-05	HPG-AHA; HPG-CTRL; MET-HPG
597.6687	28.108	1.29E-06	5.8879	4.28E-05	CTRL-AHA; HPG-AHA; MET-AHA; HPG-CTRL; MET-HPG
153.0373	27.831	1.38E-06	5.8593	4.53E-05	CTRL-AHA; HPG-AHA; HPG-CTRL; MET-HPG
571.2017	27.302	1.57E-06	5.8041	5.10E-05	CTRL-AHA; HPG-AHA; HPG-CTRL; MET-HPG
688.1455	27.088	1.65E-06	5.7815	5.33E-05	CTRL-AHA; HPG-AHA; MET-AHA; HPG-CTRL; MET-HPG
469.0623	26.878	1.74E-06	5.7591	5.56E-05	CTRL-AHA; HPG-AHA; HPG-CTRL; MET-HPG
158.0435	26.781	1.78E-06	5.7487	5.64E-05	CTRL-AHA; HPG-CTRL; MET-CTRL
311.0875	26.578	1.88E-06	5.7269	5.87E-05	CTRL-AHA; HPG-AHA; HPG-CTRL; MET-HPG
605.0014	26.55	1.89E-06	5.7239	5.87E-05	CTRL-AHA; MET-AHA; HPG-CTRL; MET-HPG
572.9464	26.481	1.92E-06	5.7166	5.92E-05	CTRL-AHA; HPG-AHA; HPG-CTRL; MET-HPG
616.6419	26.389	1.97E-06	5.7066	6.00E-05	CTRL-AHA; HPG-AHA; MET-AHA; HPG-CTRL; MET-HPG
429.1327	26.226	2.05E-06	5.6888	6.20E-05	CTRL-AHA; HPG-AHA; HPG-CTRL; MET-HPG
276.044	26.07	2.13E-06	5.6718	6.37E-05	CTRL-AHA; MET-AHA; HPG-CTRL; MET-HPG
845.2762	26.055	2.14E-06	5.6702	6.37E-05	CTRL-AHA; HPG-AHA; HPG-CTRL; MET-HPG
600.1662	25.993	2.17E-06	5.6633	6.41E-05	CTRL-AHA; HPG-AHA; MET-AHA; HPG-CTRL; MET-HPG
300.283	25.722	2.33E-06	5.6335	6.81E-05	CTRL-AHA; HPG-AHA; HPG-CTRL; MET-CTRL; MET-HPG
416.082	25.58	2.41E-06	5.6178	7.01E-05	HPG-AHA; HPG-CTRL; MET-HPG
632.115	25.311	2.58E-06	5.5878	7.45E-05	HPG-AHA; HPG-CTRL; MET-HPG
275.041	25.055	2.76E-06	5.559	7.84E-05	CTRL-AHA; MET-AHA; HPG-CTRL; MET-HPG
307.0895	25.053	2.76E-06	5.5587	7.84E-05	HPG-AHA; HPG-CTRL; MET-HPG
370.0449	24.941	2.84E-06	5.5461	8.01E-05	CTRL-AHA; HPG-AHA; HPG-CTRL; MET-HPG
686.0868	24.574	3.13E-06	5.5041	8.71E-05	CTRL-AHA; HPG-CTRL; MET-CTRL; MET-HPG
824.9636	24.561	3.14E-06	5.5027	8.71E-05	CTRL-AHA; HPG-CTRL; MET-CTRL; MET-HPG
810.6257	24.527	3.17E-06	5.4988	8.72E-05	CTRL-AHA; HPG-AHA; HPG-CTRL; MET-HPG
128.1403	24.432	3.25E-06	5.4878	8.88E-05	CTRL-AHA; MET-AHA; HPG-CTRL; MET-CTRL; MET-HPG
909.3967	24.244	3.42E-06	5.466	9.26E-05	CTRL-AHA; HPG-CTRL; MET-HPG
420.0487	24.103	3.55E-06	5.4497	9.50E-05	CTRL-AHA; MET-AHA; HPG-CTRL; MET-HPG
168.0689	24.093	3.56E-06	5.4486	9.50E-05	CTRL-AHA; HPG-AHA; MET-AHA; HPG-CTRL; MET-HPG
149.0774	24.058	3.59E-06	5.4444	9.52E-05	CTRL-AHA; HPG-CTRL; MET-HPG
225.8532	24.007	3.64E-06	5.4385	9.58E-05	CTRL-AHA; HPG-CTRL; MET-CTRL; MET-HPG
597.6691	23.978	3.67E-06	5.4351	9.58E-05	CTRL-AHA; HPG-AHA; MET-AHA; HPG-CTRL; MET-HPG
247.9936	23.916	3.73E-06	5.4278	9.67E-05	CTRL-AHA; HPG-AHA; MET-AHA; HPG-CTRL; MET-HPG
760.5372	23.869	3.78E-06	5.4222	9.73E-05	HPG-AHA; HPG-CTRL; MET-HPG
533.2459	23.801	3.85E-06	5.4143	9.84E-05	CTRL-AHA; HPG-AHA; HPG-CTRL; MET-HPG
237.0858	23.758	3.90E-06	5.4092	9.84E-05	CTRL-AHA; HPG-AHA; MET-AHA; HPG-CTRL; MET-HPG
500.0049	23.744	3.91E-06	5.4076	9.84E-05	HPG-AHA; HPG-CTRL; MET-HPG
188.1718	23.721	3.94E-06	5.4049	9.84E-05	MET-AHA; MET-CTRL; MET-HPG
307.5796	23.615	4.05E-06	5.3923	0.000101	HPG-AHA; HPG-CTRL; MET-HPG

256.0985	23.339	4.37E-06	5.3594	0.000107	CTRL-AHA; HPG-AHA; HPG-CTRL; MET-HPG
103.9531	23.336	4.37E-06	5.359	0.000107	CTRL-AHA; HPG-AHA; HPG-CTRL; MET-CTRL; MET-HPG
421.2288	23.318	4.40E-06	5.3569	0.000107	CTRL-AHA; HPG-AHA; MET-AHA; MET-CTRL
164.9909	23.203	4.54E-06	5.3431	0.00011	CTRL-AHA; HPG-CTRL; MET-CTRL; MET-HPG
293.1112	23.098	4.67E-06	5.3305	0.000111	HPG-AHA; HPG-CTRL; MET-HPG
307.1264	23.098	4.67E-06	5.3305	0.000111	HPG-AHA; HPG-CTRL; MET-HPG
811.2938	22.976	4.83E-06	5.3157	0.000114	CTRL-AHA; HPG-AHA; HPG-CTRL; MET-HPG
687.0901	22.914	4.92E-06	5.3082	0.000116	CTRL-AHA; HPG-CTRL; MET-CTRL; MET-HPG
418.9942	22.751	5.15E-06	5.2884	0.00012	HPG-AHA; MET-AHA; HPG-CTRL; MET-HPG
598.1698	22.728	5.18E-06	5.2855	0.00012	CTRL-AHA; HPG-AHA; HPG-CTRL; MET-HPG
832.9592	22.551	5.45E-06	5.2639	0.000126	CTRL-AHA; HPG-AHA; HPG-CTRL; MET-HPG
402.9468	22.433	5.63E-06	5.2492	0.000129	HPG-AHA; HPG-CTRL; MET-HPG
233.0604	22.424	5.65E-06	5.2481	0.000129	CTRL-AHA; HPG-CTRL; MET-CTRL
116.0675	22.386	5.71E-06	5.2435	0.000129	CTRL-AHA; HPG-CTRL; MET-CTRL
592.1349	22.365	5.74E-06	5.2409	0.000129	CTRL-AHA; HPG-AHA; MET-AHA
268.1231	22.334	5.79E-06	5.2371	0.000129	CTRL-AHA; HPG-CTRL; MET-CTRL; MET-HPG
175.0187	22.239	5.95E-06	5.2253	0.000132	HPG-AHA; HPG-CTRL; MET-HPG
831.9568	22.229	5.97E-06	5.224	0.000132	CTRL-AHA; HPG-CTRL; MET-CTRL
377.0975	22.102	6.19E-06	5.2082	0.000136	HPG-AHA; HPG-CTRL; MET-HPG
188.876	22.056	6.27E-06	5.2024	0.000137	CTRL-AHA; MET-AHA; HPG-CTRL; MET-HPG
259.1555	21.894	6.57E-06	5.1821	0.000142	CTRL-AHA; MET-AHA; HPG-CTRL; MET-HPG
402.2102	21.812	6.73E-06	5.1718	0.000145	MET-AHA; MET-CTRL; MET-HPG
216.954	21.681	7.00E-06	5.1552	0.00015	HPG-AHA; HPG-CTRL; MET-HPG
235.0712	21.592	7.18E-06	5.1439	0.000153	HPG-AHA; HPG-CTRL; MET-HPG
144.045	21.5	7.38E-06	5.1322	0.000156	CTRL-AHA; MET-AHA; HPG-CTRL; MET-HPG
259.0674	21.302	7.82E-06	5.1068	0.000164	CTRL-AHA; HPG-AHA; MET-AHA; HPG-CTRL; MET-HPG
319.1255	21.228	7.99E-06	5.0972	0.000167	CTRL-AHA; MET-AHA; HPG-CTRL; MET-HPG
283.258	21.139	8.21E-06	5.0856	0.000171	CTRL-AHA; HPG-CTRL; MET-CTRL; MET-HPG
192.0281	20.985	8.60E-06	5.0656	0.000178	CTRL-AHA; MET-AHA; HPG-CTRL; MET-CTRL; MET-HPG
624.6259	20.922	8.76E-06	5.0575	0.00018	CTRL-AHA; MET-AHA; HPG-CTRL; MET-HPG
320.7104	20.825	9.02E-06	5.0447	0.000184	CTRL-AHA; HPG-CTRL; MET-CTRL
241.1794	20.734	9.27E-06	5.0328	0.000188	CTRL-AHA; MET-AHA; HPG-CTRL; MET-HPG
656.8367	20.669	9.46E-06	5.0243	0.000191	HPG-AHA; HPG-CTRL; MET-HPG
173.039	20.621	9.59E-06	5.018	0.000193	HPG-AHA; HPG-CTRL; MET-HPG
591.1312	20.523	9.89E-06	5.005	0.000197	CTRL-AHA; HPG-AHA; MET-AHA; HPG-CTRL
708.0687	20.301	1.06E-05	4.9754	0.00021	CTRL-AHA; MET-AHA; HPG-CTRL; MET-HPG
704.0622	20.194	1.09E-05	4.9611	0.000216	CTRL-AHA; MET-AHA; HPG-CTRL; MET-HPG
794.486	19.874	1.21E-05	4.9179	0.000237	CTRL-AHA; HPG-AHA; HPG-CTRL; MET-HPG
457.2414	19.733	1.26E-05	4.8987	0.000247	CTRL-AHA; HPG-AHA; MET-AHA; HPG-CTRL; MET-HPG
310.0818	19.707	1.27E-05	4.8951	0.000247	CTRL-AHA; HPG-CTRL; MET-CTRL; MET-HPG
766.9187	19.566	1.33E-05	4.8759	0.000257	HPG-AHA; HPG-CTRL; MET-HPG
307.5797	19.491	1.36E-05	4.8655	0.000262	HPG-AHA; MET-AHA; HPG-CTRL; MET-HPG
306.1902	19.469	1.37E-05	4.8626	0.000262	CTRL-AHA; HPG-AHA; MET-CTRL; MET-HPG
851.9494	19.432	1.39E-05	4.8573	0.000264	CTRL-AHA; HPG-CTRL; MET-CTRL
147.1094	19.337	1.43E-05	4.8443	0.000271	HPG-AHA; HPG-CTRL; MET-HPG
96.04388	19.317	1.44E-05	4.8415	0.000271	HPG-AHA; HPG-CTRL; MET-HPG
823.6105	19.266	1.46E-05	4.8343	0.000274	CTRL-AHA; HPG-AHA; HPG-CTRL; MET-HPG
385.1121	19.196	1.50E-05	4.8247	0.000278	HPG-AHA; HPG-CTRL; MET-HPG
189.1746	19.193	1.50E-05	4.8242	0.000278	MET-AHA; MET-CTRL; MET-HPG
480.9936	19.142	1.52E-05	4.8171	0.000281	HPG-AHA; HPG-CTRL; MET-HPG
221.0254	19.105	1.54E-05	4.8119	0.000282	CTRL-AHA; HPG-CTRL; MET-HPG
162.0182	19.095	1.55E-05	4.8104	0.000282	CTRL-AHA; HPG-AHA; HPG-CTRL; MET-HPG
402.0125	19.013	1.59E-05	4.7989	0.000288	CTRL-AHA; HPG-AHA; HPG-CTRL; MET-HPG
171.1452	18.894	1.65E-05	4.7822	0.000298	MET-AHA; MET-CTRL; MET-HPG
439.0625	18.751	1.73E-05	4.762	0.000311	CTRL-AHA; HPG-CTRL; MET-CTRL
606.1243	18.684	1.77E-05	4.7524	0.000316	HPG-AHA; HPG-CTRL; MET-HPG
367.0533	18.626	1.80E-05	4.7441	0.000321	HPG-AHA; HPG-CTRL; MET-HPG
921.9952	18.503	1.88E-05	4.7266	0.000332	CTRL-AHA; HPG-CTRL; MET-CTRL; MET-HPG
283.258	18.439	1.92E-05	4.7174	0.000338	CTRL-AHA; HPG-CTRL; MET-CTRL
173.5405	18.301	2.01E-05	4.6975	0.000352	HPG-AHA; HPG-CTRL; MET-HPG
180.0476	18.242	2.05E-05	4.6889	0.000357	CTRL-AHA; HPG-CTRL; MET-CTRL; MET-HPG
758.2555	18.198	2.08E-05	4.6826	0.00036	CTRL-AHA; MET-AHA; HPG-CTRL; MET-HPG
382.9945	18.147	2.11E-05	4.6751	0.000365	CTRL-AHA; MET-AHA; HPG-CTRL; MET-HPG
456.2377	18.126	2.13E-05	4.6721	0.000365	CTRL-AHA; MET-AHA; HPG-CTRL; MET-HPG
102.1253	18.118	2.13E-05	4.6708	0.000365	CTRL-AHA; MET-AHA; HPG-CTRL; MET-HPG
369.1177	18.095	2.15E-05	4.6676	0.000366	HPG-AHA; HPG-CTRL; MET-HPG
145.047	18.021	2.20E-05	4.6567	0.000373	CTRL-AHA; MET-AHA; HPG-CTRL; MET-CTRL; MET-HPG
520.9049	17.986	2.23E-05	4.6516	0.000376	CTRL-AHA; HPG-AHA; HPG-CTRL; MET-HPG
832.2904	17.952	2.26E-05	4.6466	0.000379	HPG-AHA; HPG-CTRL; MET-HPG
171.1817	17.856	2.33E-05	4.6325	0.000389	CTRL-AHA; MET-AHA; HPG-CTRL; MET-HPG
132.099	17.662	2.49E-05	4.6037	0.000414	CTRL-AHA; HPG-CTRL; MET-CTRL
616.6419	17.517	2.62E-05	4.5821	0.000432	CTRL-AHA; HPG-AHA; MET-AHA; HPG-CTRL; MET-HPG
100.1096	17.509	2.62E-05	4.5809	0.000432	CTRL-AHA; HPG-AHA; MET-AHA
655.1169	17.474	2.66E-05	4.5758	0.000436	HPG-AHA; HPG-CTRL; MET-HPG

272.1374	17.46	2.67E-05	4.5736	0.000436	CTRL-AHA; MET-AHA; HPG-CTRL; MET-HPG
118.0835	17.435	2.69E-05	4.5699	0.000437	CTRL-AHA; HPG-AHA; HPG-CTRL; MET-CTRL
482.9484	17.388	2.74E-05	4.5627	0.000443	HPG-AHA; HPG-CTRL; MET-HPG
242.0615	17.332	2.79E-05	4.5543	0.000449	CTRL-AHA; MET-AHA; HPG-CTRL; MET-HPG
141.0013	17.312	2.81E-05	4.5513	0.000449	CTRL-AHA; HPG-CTRL; MET-HPG
289.0438	17.311	2.81E-05	4.5511	0.000449	HPG-AHA; HPG-CTRL; MET-HPG
845.9586	17.29	2.83E-05	4.5481	0.00045	CTRL-AHA; HPG-CTRL; MET-CTRL
615.1509	17.2	2.92E-05	4.5344	0.000462	CTRL-AHA; HPG-AHA; HPG-CTRL; MET-HPG
873.2821	17.069	3.06E-05	4.5145	0.00048	HPG-AHA; HPG-CTRL; MET-HPG
373.0805	17.064	3.06E-05	4.5138	0.00048	CTRL-AHA; MET-AHA; HPG-CTRL; MET-HPG
846.2929	17.046	3.08E-05	4.511	0.000481	CTRL-AHA; HPG-CTRL; MET-CTRL
473.1627	16.994	3.14E-05	4.503	0.000488	CTRL-AHA; MET-AHA; HPG-CTRL; MET-HPG
132.099	16.882	3.27E-05	4.4858	0.000506	CTRL-AHA; HPG-CTRL; MET-CTRL
394.9969	16.829	3.33E-05	4.4777	0.000513	HPG-AHA; HPG-CTRL; MET-HPG
825.2973	16.783	3.38E-05	4.4706	0.000519	HPG-AHA; HPG-CTRL; MET-HPG
764.6054	16.71	3.47E-05	4.4594	0.000531	CTRL-AHA; HPG-CTRL; MET-CTRL
402.8166	16.675	3.52E-05	4.454	0.000535	CTRL-AHA; HPG-CTRL; MET-CTRL; MET-HPG
169.0786	16.651	3.55E-05	4.4501	0.000537	CTRL-AHA; MET-AHA; HPG-CTRL; MET-HPG
405.0025	16.621	3.59E-05	4.4455	0.000541	CTRL-AHA; HPG-CTRL; MET-HPG
72.0791	16.486	3.76E-05	4.4245	0.000565	CTRL-AHA; HPG-CTRL; MET-HPG
383.0236	16.29	4.04E-05	4.3936	0.000604	HPG-AHA; HPG-CTRL; MET-HPG
479.0816	16.262	4.08E-05	4.3892	0.000608	HPG-AHA; HPG-CTRL; MET-HPG
405.0024	16.197	4.18E-05	4.3789	0.000617	CTRL-AHA; HPG-CTRL; MET-HPG
260.0701	16.19	4.19E-05	4.3778	0.000617	HPG-AHA; HPG-CTRL; MET-HPG
372.9554	16.187	4.19E-05	4.3774	0.000617	HPG-AHA; HPG-CTRL; MET-HPG
607.1046	16.119	4.30E-05	4.3665	0.00063	CTRL-AHA; MET-AHA; HPG-CTRL
824.6286	16.077	4.37E-05	4.3599	0.000636	CTRL-AHA; HPG-CTRL; MET-CTRL
636.4502	16.066	4.38E-05	4.3581	0.000636	HPG-AHA; HPG-CTRL; MET-HPG
304.1808	16.059	4.40E-05	4.357	0.000636	CTRL-AHA; HPG-AHA; HPG-CTRL; MET-CTRL
420.2265	15.999	4.49E-05	4.3474	0.000648	CTRL-AHA; HPG-AHA; MET-CTRL; MET-HPG
823.2758	15.921	4.62E-05	4.3349	0.000664	HPG-AHA; HPG-CTRL; MET-HPG
489.9245	15.911	4.64E-05	4.3332	0.000664	CTRL-AHA; HPG-CTRL; MET-HPG
648.2625	15.802	4.83E-05	4.3158	0.000688	CTRL-AHA; HPG-CTRL; MET-HPG
307.0785	15.779	4.87E-05	4.3121	0.000691	HPG-AHA; HPG-CTRL; MET-HPG
157.0418	15.709	5.00E-05	4.3007	0.000707	CTRL-AHA; MET-AHA; HPG-CTRL
750.9434	15.631	5.15E-05	4.288	0.000725	HPG-AHA; HPG-CTRL; MET-HPG
771.5548	15.606	5.20E-05	4.284	0.000729	CTRL-AHA; HPG-CTRL; MET-CTRL
433.1229	15.561	5.29E-05	4.2766	0.000739	HPG-AHA; HPG-CTRL; MET-HPG
192.0272	15.512	5.39E-05	4.2687	0.000749	CTRL-AHA; MET-AHA; HPG-CTRL; MET-CTRL
617.1651	15.499	5.41E-05	4.2665	0.00075	CTRL-AHA; HPG-AHA; HPG-CTRL; MET-HPG
352.2203	15.453	5.51E-05	4.2589	0.00076	CTRL-AHA; MET-AHA; HPG-CTRL
277.0402	15.431	5.55E-05	4.2554	0.000764	CTRL-AHA; MET-AHA; HPG-CTRL; MET-HPG
411.1067	15.42	5.58E-05	4.2535	0.000764	CTRL-AHA; HPG-CTRL; MET-HPG
446.0681	15.323	5.79E-05	4.2375	0.00079	HPG-AHA; HPG-CTRL; MET-HPG
832.2893	15.255	5.94E-05	4.2262	0.000807	CTRL-AHA; HPG-CTRL; MET-CTRL
174.0385	15.066	6.39E-05	4.1948	0.000865	HPG-AHA; HPG-CTRL; MET-HPG
859.9611	15.017	6.51E-05	4.1867	0.000878	CTRL-AHA; HPG-CTRL; MET-CTRL
449.7817	14.993	6.57E-05	4.1827	0.000882	CTRL-AHA; HPG-CTRL; MET-CTRL
129.9289	14.98	6.60E-05	4.1805	0.000884	CTRL-AHA; HPG-CTRL; MET-CTRL
724.9749	14.97	6.62E-05	4.1789	0.000884	HPG-AHA; HPG-CTRL; MET-HPG
832.6236	14.961	6.65E-05	4.1772	0.000884	HPG-AHA; HPG-CTRL; MET-HPG
370.2523	14.922	6.75E-05	4.1707	0.000894	CTRL-AHA; HPG-CTRL; MET-CTRL
402.0676	14.845	6.95E-05	4.1577	0.000917	CTRL-AHA; HPG-CTRL; MET-HPG
665.1087	14.79	7.10E-05	4.1485	0.000934	CTRL-AHA; HPG-CTRL; MET-CTRL
378.2164	14.702	7.35E-05	4.1335	0.000963	CTRL-AHA; HPG-AHA; MET-AHA; HPG-CTRL; MET-CTRL
397.0897	14.687	7.40E-05	4.131	0.000965	HPG-AHA; HPG-CTRL; MET-HPG
189.1746	14.59	7.68E-05	4.1145	0.000997	MET-AHA; MET-CTRL; MET-HPG
534.8978	14.586	7.70E-05	4.1138	0.000997	CTRL-AHA; HPG-CTRL; MET-HPG
636.1335	14.571	7.74E-05	4.1112	0.000999	HPG-AHA; HPG-CTRL; MET-HPG
156.0386	14.549	7.81E-05	4.1075	0.001004	CTRL-AHA; MET-AHA; HPG-CTRL
116.0676	14.536	7.85E-05	4.1051	0.001006	CTRL-AHA; HPG-CTRL; MET-CTRL
665.4842	14.515	7.92E-05	4.1015	0.001008	HPG-AHA; HPG-CTRL; MET-HPG
979.5301	14.512	7.92E-05	4.1011	0.001008	CTRL-AHA; HPG-AHA; HPG-CTRL; MET-HPG
692.1309	14.471	8.05E-05	4.0941	0.001021	HPG-AHA; HPG-CTRL; MET-HPG
418.0747	14.45	8.12E-05	4.0904	0.001026	HPG-AHA; HPG-CTRL; MET-HPG
481.2328	14.393	8.31E-05	4.0806	0.001046	CTRL-AHA; MET-AHA; HPG-CTRL; MET-HPG
360.1707	14.362	8.41E-05	4.0752	0.001055	CTRL-AHA; HPG-CTRL; MET-CTRL
438.1669	14.342	8.48E-05	4.0718	0.00106	HPG-AHA; HPG-CTRL; MET-HPG
417.082	14.21	8.94E-05	4.0489	0.001113	HPG-AHA; HPG-CTRL; MET-HPG
200.9836	14.123	9.25E-05	4.0337	0.001149	HPG-AHA; HPG-CTRL; MET-HPG
564.0406	14.104	9.33E-05	4.0303	0.001154	CTRL-AHA; HPG-CTRL; MET-CTRL
73.04166	14.076	9.43E-05	4.0255	0.001163	HPG-AHA; HPG-CTRL; MET-HPG
321.1064	14.037	9.58E-05	4.0185	0.001177	HPG-AHA; HPG-CTRL; MET-HPG
372.077	13.951	9.92E-05	4.0034	0.001215	HPG-AHA; HPG-CTRL; MET-HPG

678.4901	13.925	0.0001	3.9988	0.001224	HPG-AHA; HPG-CTRL; MET-HPG
559.9045	13.829	0.000104	3.9819	0.001268	CTRL-AHA; HPG-CTRL; MET-HPG
394.9508	13.694	0.00011	3.9577	0.001336	HPG-AHA; HPG-CTRL; MET-HPG
466.1084	13.68	0.000111	3.9553	0.001339	HPG-AHA; HPG-CTRL; MET-HPG
462.1224	13.622	0.000114	3.9449	0.001367	CTRL-AHA; HPG-AHA; MET-AHA; MET-CTRL
508.1504	13.554	0.000117	3.9326	0.0014	CTRL-AHA; HPG-CTRL; MET-HPG
784.0531	13.547	0.000117	3.9315	0.0014	CTRL-AHA; HPG-CTRL; MET-HPG
421.935	13.535	0.000118	3.9292	0.001403	HPG-AHA; HPG-CTRL; MET-HPG
219.0755	13.474	0.000121	3.9183	0.001434	CTRL-AHA; HPG-CTRL; MET-CTRL
423.1836	13.375	0.000126	3.9002	0.00149	MET-AHA; HPG-CTRL; MET-HPG
757.7545	13.365	0.000126	3.8984	0.001491	CTRL-AHA; MET-AHA; HPG-CTRL; MET-HPG
536.8797	13.352	0.000127	3.8961	0.001494	CTRL-AHA; HPG-AHA; HPG-CTRL; MET-HPG
399.9712	13.298	0.00013	3.8863	0.001523	CTRL-AHA; HPG-AHA; HPG-CTRL; MET-HPG
491.8094	13.273	0.000131	3.8817	0.001534	CTRL-AHA; HPG-CTRL; MET-CTRL
676.4811	13.258	0.000132	3.8789	0.001539	HPG-AHA; HPG-CTRL; MET-HPG
339.1708	13.247	0.000133	3.877	0.001541	CTRL-AHA; HPG-AHA; MET-HPG
143.1508	13.217	0.000134	3.8715	0.001555	CTRL-AHA; HPG-CTRL; MET-CTRL
163.057	13.122	0.00014	3.8539	0.001614	CTRL-AHA; HPG-CTRL; MET-CTRL; MET-HPG
818.6205	13.113	0.000141	3.8523	0.001615	HPG-AHA; HPG-CTRL; MET-HPG
587.027	13.097	0.000141	3.8494	0.001621	CTRL-AHA; MET-AHA; HPG-CTRL; MET-HPG
312.1085	13.061	0.000144	3.8427	0.001641	CTRL-AHA; HPG-AHA; MET-CTRL
702.4954	12.924	0.000152	3.8174	0.001734	HPG-AHA; HPG-CTRL; MET-HPG
465.0275	12.81	0.00016	3.796	0.001816	HPG-AHA; HPG-CTRL; MET-HPG
143.1508	12.785	0.000162	3.7914	0.001829	CTRL-AHA; HPG-CTRL; MET-HPG
589.0333	12.776	0.000162	3.7897	0.00183	HPG-AHA; HPG-CTRL; MET-HPG
400.9488	12.752	0.000164	3.7851	0.001844	HPG-AHA; HPG-CTRL; MET-HPG
307.0789	12.731	0.000166	3.7812	0.001853	HPG-AHA; HPG-CTRL; MET-HPG
455.2523	12.726	0.000166	3.7803	0.001853	HPG-AHA; HPG-CTRL; MET-HPG
345.0401	12.706	0.000167	3.7765	0.001863	CTRL-AHA; MET-AHA; HPG-CTRL; MET-HPG
677.4844	12.684	0.000169	3.7724	0.001873	HPG-AHA; HPG-CTRL; MET-HPG
186.2175	12.679	0.000169	3.7715	0.001873	HPG-AHA; HPG-CTRL; MET-HPG
319.1116	12.67	0.00017	3.7696	0.001873	HPG-AHA; HPG-CTRL; MET-HPG
420.0489	12.665	0.00017	3.7688	0.001873	CTRL-AHA; MET-AHA; HPG-CTRL
143.0782	12.615	0.000174	3.7593	0.001903	CTRL-AHA; MET-AHA; HPG-CTRL
387.0203	12.614	0.000174	3.7592	0.001903	HPG-AHA; HPG-CTRL; MET-HPG
618.8803	12.606	0.000175	3.7576	0.001905	HPG-AHA; HPG-CTRL; MET-HPG
368.005	12.592	0.000176	3.7549	0.001905	HPG-AHA; HPG-CTRL; MET-HPG
76.0197	12.585	0.000176	3.7536	0.001905	CTRL-AHA; HPG-CTRL; MET-HPG
702.0786	12.585	0.000176	3.7536	0.001905	CTRL-AHA; HPG-CTRL; MET-HPG
285.9626	12.565	0.000178	3.7499	0.001916	CTRL-AHA; HPG-AHA; HPG-CTRL; MET-HPG
176.0993	12.543	0.00018	3.7457	0.001925	HPG-AHA; HPG-CTRL; MET-HPG
846.6265	12.54	0.00018	3.7452	0.001925	CTRL-AHA; HPG-CTRL; MET-CTRL
472.2145	12.492	0.000184	3.7359	0.00196	CTRL-AHA; MET-AHA; HPG-CTRL; MET-HPG
572.9469	12.438	0.000188	3.7256	0.002001	HPG-AHA; HPG-CTRL; MET-HPG
477.2414	12.327	0.000198	3.7044	0.002095	MET-AHA; MET-CTRL; MET-HPG
403.0226	12.282	0.000201	3.6958	0.002131	CTRL-AHA; MET-AHA; HPG-CTRL
326.0417	12.266	0.000203	3.6926	0.00214	HPG-AHA; HPG-CTRL; MET-HPG
378.9783	12.236	0.000206	3.6868	0.002163	HPG-AHA; HPG-CTRL; MET-HPG
569.1498	12.174	0.000211	3.6749	0.002217	CTRL-AHA; MET-AHA; HPG-CTRL
134.0416	12.163	0.000212	3.6727	0.002221	MET-AHA; HPG-CTRL; MET-HPG
588.9191	12.142	0.000214	3.6686	0.002235	HPG-AHA; HPG-CTRL; MET-HPG
560.1328	12.132	0.000215	3.6667	0.002239	HPG-AHA; HPG-CTRL; MET-HPG
232.02	12.107	0.000218	3.6618	0.002258	CTRL-AHA; MET-AHA; HPG-CTRL; MET-HPG
200.9833	12.068	0.000222	3.6542	0.002291	HPG-AHA; HPG-CTRL; MET-HPG
241.2174	12.049	0.000224	3.6505	0.002304	CTRL-AHA; MET-AHA; HPG-CTRL; MET-HPG
890.7436	12.026	0.000226	3.6461	0.002321	CTRL-AHA; HPG-CTRL; MET-HPG
980.0314	12.018	0.000227	3.6446	0.002322	CTRL-AHA; HPG-CTRL; MET-HPG
258.1457	11.928	0.000236	3.6269	0.002412	CTRL-AHA; HPG-AHA; MET-AHA
372.0315	11.887	0.000241	3.6189	0.00245	HPG-AHA; HPG-CTRL; MET-HPG
268.1477	11.832	0.000247	3.6079	0.002505	CTRL-AHA; HPG-CTRL; MET-CTRL
766.916	11.795	0.000251	3.6006	0.00254	HPG-AHA; HPG-CTRL; MET-HPG
632.8737	11.745	0.000257	3.5908	0.002583	CTRL-AHA; HPG-CTRL; MET-HPG
258.6563	11.741	0.000257	3.59	0.002583	CTRL-AHA; HPG-CTRL; MET-HPG
161.0984	11.74	0.000257	3.5897	0.002583	CTRL-AHA; HPG-CTRL; MET-HPG
242.0613	11.711	0.000261	3.5839	0.00261	CTRL-AHA; HPG-CTRL; MET-HPG
672.8103	11.7	0.000262	3.5817	0.002616	HPG-AHA; HPG-CTRL; MET-HPG
240.1545	11.689	0.000263	3.5796	0.002622	CTRL-AHA; HPG-CTRL; MET-HPG
846.961	11.669	0.000266	3.5757	0.002638	CTRL-AHA; HPG-CTRL; MET-HPG
818.2865	11.631	0.00027	3.568	0.002678	HPG-AHA; HPG-CTRL; MET-HPG
239.0871	11.546	0.000281	3.551	0.002777	MET-AHA; MET-CTRL; MET-HPG
664.4804	11.512	0.000286	3.5441	0.002814	HPG-AHA; HPG-CTRL; MET-HPG
486.0867	11.49	0.000289	3.5398	0.002834	CTRL-AHA; MET-AHA; HPG-CTRL
363.9966	11.441	0.000295	3.5299	0.002891	HPG-AHA; HPG-CTRL; MET-HPG
653.1198	11.418	0.000298	3.5251	0.002915	CTRL-AHA; HPG-CTRL; MET-HPG

767.5631	11.386	0.000303	3.5188	0.002934	CTRL-AHA; HPG-CTRL; MET-CTRL
272.0926	11.384	0.000303	3.5183	0.002934	CTRL-AHA; HPG-AHA; MET-HPG
257.1434	11.38	0.000304	3.5174	0.002934	CTRL-AHA; HPG-AHA; MET-AHA
549.4793	11.376	0.000304	3.5167	0.002934	HPG-AHA; HPG-CTRL; MET-HPG
262.7527	11.375	0.000304	3.5165	0.002934	CTRL-AHA; HPG-CTRL; MET-CTRL
519.2645	11.334	0.00031	3.5083	0.002976	HPG-AHA; HPG-CTRL; MET-HPG
652.1088	11.327	0.000311	3.5068	0.002976	HPG-AHA; HPG-CTRL; MET-HPG
158.0433	11.327	0.000311	3.5068	0.002976	CTRL-AHA; HPG-CTRL; MET-CTRL
654.8548	11.319	0.000313	3.5051	0.002979	CTRL-AHA; HPG-CTRL; MET-CTRL
297.0226	11.312	0.000314	3.5036	0.002982	HPG-CTRL; MET-HPG
844.6069	11.24	0.000324	3.4891	0.003075	HPG-AHA; HPG-CTRL; MET-HPG
235.1606	11.198	0.000331	3.4805	0.003128	HPG-AHA; HPG-CTRL; MET-HPG
664.1059	11.191	0.000332	3.4789	0.003131	CTRL-AHA; HPG-CTRL
386.2023	11.148	0.000339	3.4702	0.003186	CTRL-AHA; HPG-CTRL; MET-HPG
584.9771	11.136	0.000341	3.4677	0.003194	CTRL-AHA; HPG-CTRL; MET-HPG
242.0614	11.125	0.000342	3.4655	0.003194	HPG-AHA; HPG-CTRL; MET-HPG
689.1392	11.123	0.000343	3.4651	0.003194	HPG-AHA; HPG-CTRL; MET-HPG
173.0168	11.121	0.000343	3.4646	0.003194	CTRL-AHA; HPG-CTRL; MET-CTRL
600.9523	11.11	0.000345	3.4623	0.003203	CTRL-AHA; HPG-CTRL; MET-HPG
838.281	11.09	0.000348	3.4582	0.003224	CTRL-AHA; HPG-CTRL; MET-HPG
402.2099	11.045	0.000356	3.4489	0.003283	MET-AHA; MET-CTRL; MET-HPG
348.8508	11.041	0.000356	3.4482	0.003283	CTRL-AHA; HPG-CTRL; MET-CTRL
240.1761	11.025	0.000359	3.4448	0.003299	CTRL-AHA; MET-AHA; HPG-CTRL; MET-HPG
703.0635	11.02	0.00036	3.4438	0.003299	CTRL-AHA; HPG-CTRL; MET-HPG
73.08239	10.995	0.000364	3.4385	0.003331	CTRL-AHA; HPG-CTRL
822.015	10.951	0.000372	3.4295	0.003392	CTRL-AHA; HPG-CTRL; MET-HPG
172.039	10.919	0.000378	3.4228	0.003436	CTRL-AHA; HPG-CTRL; MET-HPG
144.0621	10.903	0.000381	3.4194	0.003455	CTRL-AHA; MET-AHA; HPG-CTRL; MET-HPG
825.2962	10.882	0.000385	3.4151	0.003464	CTRL-AHA; HPG-CTRL; MET-HPG
462.7409	10.877	0.000385	3.4141	0.003464	CTRL-AHA; MET-AHA; HPG-CTRL
479.136	10.874	0.000386	3.4135	0.003464	CTRL-AHA; MET-AHA; HPG-CTRL; MET-HPG
403.0227	10.874	0.000386	3.4133	0.003464	CTRL-AHA; MET-AHA; HPG-CTRL
184.1483	10.871	0.000387	3.4128	0.003464	CTRL-AHA; MET-AHA; MET-HPG
219.076	10.866	0.000388	3.4116	0.003464	CTRL-AHA; HPG-CTRL; MET-CTRL
301.9367	10.846	0.000391	3.4076	0.003487	HPG-AHA; HPG-CTRL; MET-HPG
238.089	10.824	0.000395	3.4029	0.003517	HPG-AHA; HPG-CTRL
329.1077	10.813	0.000397	3.4007	0.003526	HPG-CTRL; MET-HPG
469.0622	10.8	0.0004	3.3979	0.00354	HPG-AHA; HPG-CTRL; MET-HPG
312.1117	10.788	0.000402	3.3954	0.003551	CTRL-AHA; HPG-AHA; MET-CTRL; MET-HPG
973.3348	10.755	0.000409	3.3886	0.003599	CTRL-AHA; MET-AHA; HPG-CTRL
244.0579	10.728	0.000414	3.3828	0.003638	CTRL-AHA; HPG-CTRL; MET-CTRL
667.1098	10.692	0.000421	3.3752	0.003693	CTRL-AHA; HPG-CTRL
556.2098	10.687	0.000423	3.3741	0.003694	CTRL-AHA; MET-AHA; HPG-CTRL
478.2439	10.681	0.000424	3.3728	0.003696	MET-AHA; MET-CTRL; MET-HPG
307.0801	10.667	0.000427	3.37	0.003711	HPG-AHA; HPG-CTRL; MET-HPG
615.1638	10.63	0.000434	3.3622	0.003769	CTRL-AHA; HPG-CTRL; MET-HPG
410.924	10.598	0.000441	3.3552	0.003821	HPG-AHA; HPG-CTRL; MET-HPG
497.1464	10.537	0.000455	3.3422	0.003918	HPG-CTRL; MET-HPG
312.9957	10.535	0.000455	3.3419	0.003918	HPG-AHA; HPG-CTRL; MET-HPG
396.1521	10.528	0.000457	3.3404	0.003918	HPG-AHA; HPG-CTRL; MET-HPG
532.9632	10.527	0.000457	3.3401	0.003918	HPG-AHA; HPG-CTRL; MET-HPG
196.0934	10.514	0.00046	3.3374	0.003925	HPG-CTRL; MET-HPG
175.1397	10.513	0.00046	3.3372	0.003925	CTRL-AHA; HPG-CTRL; MET-HPG
174.8918	10.499	0.000463	3.3341	0.003944	CTRL-AHA; HPG-CTRL; MET-CTRL
72.93538	10.477	0.000468	3.3295	0.003976	CTRL-AHA; HPG-CTRL; MET-HPG
160.0841	10.469	0.00047	3.3277	0.003983	CTRL-AHA; HPG-CTRL; MET-CTRL
634.8554	10.46	0.000472	3.3258	0.003991	HPG-CTRL; MET-HPG
388.0505	10.448	0.000475	3.3233	0.004005	HPG-CTRL; MET-HPG
670.8278	10.402	0.000486	3.3134	0.004087	HPG-CTRL; MET-HPG
852.2834	10.374	0.000493	3.3075	0.004134	HPG-AHA; HPG-CTRL; MET-HPG
193.0313	10.322	0.000506	3.2962	0.004233	CTRL-AHA; HPG-AHA; MET-AHA
162.0547	10.302	0.000511	3.2918	0.004257	HPG-AHA; HPG-CTRL
124.084	10.298	0.000512	3.291	0.004257	CTRL-AHA; HPG-CTRL; MET-HPG
464.9768	10.297	0.000512	3.2907	0.004257	HPG-AHA; HPG-CTRL; MET-HPG
498.9219	10.29	0.000514	3.2893	0.004261	HPG-AHA; HPG-CTRL; MET-HPG
302.077	10.277	0.000517	3.2864	0.004279	CTRL-AHA; HPG-AHA; MET-AHA
214.0721	10.248	0.000524	3.2803	0.00433	HPG-AHA; MET-CTRL; MET-HPG
180.0481	10.225	0.000531	3.2752	0.004371	CTRL-AHA; MET-AHA; HPG-CTRL; MET-HPG
487.158	10.214	0.000534	3.2727	0.00438	CTRL-AHA; HPG-CTRL
770.5178	10.212	0.000534	3.2724	0.00438	CTRL-AHA; HPG-CTRL
858.9457	10.204	0.000536	3.2708	0.004386	CTRL-AHA; HPG-CTRL; MET-HPG
837.4282	10.185	0.000541	3.2666	0.004418	CTRL-AHA; HPG-AHA
618.1666	10.152	0.00055	3.2594	0.004482	CTRL-AHA; HPG-CTRL; MET-HPG
541.9353	10.13	0.000556	3.2546	0.004521	CTRL-AHA; HPG-CTRL; MET-HPG

112.073	10.111	0.000562	3.2503	0.004556	CTRL-AHA; MET-AHA
222.1034	10.073	0.000573	3.242	0.004633	HPG-CTRL; MET-HPG
362.0105	10.056	0.000578	3.2384	0.004661	HPG-AHA; HPG-CTRL; MET-HPG
393.1449	9.9636	0.000605	3.218	0.004874	CTRL-AHA; HPG-CTRL; MET-HPG
403.0227	9.8487	0.000642	3.1925	0.005149	CTRL-AHA; MET-AHA; HPG-CTRL
326.1106	9.8475	0.000642	3.1923	0.005149	CTRL-AHA; HPG-CTRL; MET-CTRL
768.5247	9.8376	0.000646	3.1901	0.005163	CTRL-AHA; HPG-CTRL; MET-CTRL
570.1556	9.804	0.000657	3.1826	0.005241	CTRL-AHA; MET-AHA
769.5601	9.795	0.00066	3.1806	0.005254	CTRL-AHA; HPG-CTRL
215.0021	9.7886	0.000662	3.1791	0.005255	CTRL-AHA; HPG-CTRL; MET-HPG
325.1071	9.7858	0.000663	3.1785	0.005255	CTRL-AHA; HPG-CTRL
413.9823	9.7518	0.000675	3.1709	0.005325	HPG-AHA; HPG-CTRL; MET-HPG
820.5198	9.7517	0.000675	3.1709	0.005325	HPG-AHA; HPG-CTRL; MET-HPG
853.9633	9.7434	0.000678	3.169	0.005336	CTRL-AHA; HPG-CTRL; MET-HPG
146.0351	9.731	0.000682	3.1662	0.005343	CTRL-AHA; MET-AHA; HPG-CTRL
847.295	9.7301	0.000682	3.166	0.005343	HPG-CTRL; MET-HPG
144.0447	9.728	0.000683	3.1656	0.005343	CTRL-AHA; MET-AHA; HPG-CTRL; MET-HPG
160.0492	9.7095	0.00069	3.1614	0.005383	CTRL-AHA; HPG-CTRL
776.5119	9.6648	0.000706	3.1514	0.005496	CTRL-AHA; HPG-CTRL
550.2891	9.6603	0.000707	3.1504	0.005497	CTRL-AHA; HPG-CTRL; MET-HPG
391.2776	9.6297	0.000719	3.1435	0.005573	CTRL-AHA; HPG-CTRL; MET-CTRL
58.06367	9.6235	0.000721	3.1421	0.005579	CTRL-AHA; HPG-CTRL; MET-CTRL
259.0171	9.5905	0.000733	3.1346	0.005645	HPG-AHA; HPG-CTRL; MET-HPG
364.9927	9.5833	0.000736	3.133	0.005645	HPG-AHA; HPG-CTRL; MET-HPG
716.5119	9.5808	0.000737	3.1325	0.005645	HPG-AHA; HPG-CTRL; MET-HPG
227.0281	9.5806	0.000737	3.1324	0.005645	CTRL-AHA; HPG-CTRL; MET-CTRL
703.4991	9.5801	0.000737	3.1323	0.005645	HPG-CTRL; MET-HPG
234.0572	9.5598	0.000745	3.1277	0.005693	HPG-CTRL; MET-HPG
851.4414	9.5447	0.000751	3.1243	0.005726	CTRL-AHA; HPG-CTRL; MET-HPG
516.9894	9.5295	0.000757	3.1208	0.005759	CTRL-AHA; HPG-CTRL
353.1575	9.4481	0.00079	3.1023	0.005997	HPG-AHA; HPG-CTRL; MET-HPG
864.6026	9.4204	0.000802	3.096	0.006059	CTRL-AHA; HPG-CTRL; MET-HPG
158.9182	9.4204	0.000802	3.096	0.006059	CTRL-AHA; HPG-CTRL; MET-CTRL
310.0832	9.3981	0.000811	3.0909	0.006118	HPG-CTRL; MET-HPG
306.1902	9.3842	0.000817	3.0877	0.00615	HPG-AHA; MET-HPG
400.8204	9.3432	0.000835	3.0783	0.006271	CTRL-AHA; HPG-CTRL; MET-HPG
609.9229	9.2968	0.000856	3.0676	0.006414	CTRL-AHA; HPG-CTRL; MET-HPG
244.0742	9.2746	0.000866	3.0625	0.006476	CTRL-AHA; HPG-CTRL
131.1148	9.2639	0.000871	3.06	0.0065	CTRL-AHA; HPG-AHA; MET-AHA
673.087	9.2541	0.000875	3.0578	0.006515	HPG-AHA; HPG-CTRL; MET-HPG
305.1595	9.2516	0.000877	3.0572	0.006515	CTRL-AHA; HPG-CTRL; MET-HPG
284.3249	9.2419	0.000881	3.055	0.006535	HPG-CTRL; MET-HPG
224.0875	9.2	0.000901	3.0453	0.006669	MET-AHA; MET-HPG
211.0492	9.1508	0.000925	3.0338	0.006833	CTRL-AHA; HPG-AHA; MET-HPG
879.9437	9.1417	0.00093	3.0317	0.006852	HPG-CTRL; MET-HPG
145.1665	9.1053	0.000948	3.0232	0.006973	CTRL-AHA; HPG-CTRL
163.0755	9.097	0.000952	3.0213	0.006989	HPG-AHA; HPG-CTRL; MET-HPG
449.0029	9.088	0.000957	3.0192	0.006998	CTRL-AHA; HPG-CTRL; MET-HPG
385.6999	9.0871	0.000957	3.019	0.006998	CTRL-AHA; HPG-CTRL; MET-HPG
873.6133	9.0676	0.000967	3.0144	0.007057	CTRL-AHA; HPG-CTRL; MET-HPG
425.973	9.0512	0.000976	3.0106	0.007105	HPG-CTRL; MET-HPG
413.157	9.0269	0.000989	3.0049	0.007184	HPG-AHA; HPG-CTRL; MET-HPG
617.054	9.0122	0.000997	3.0015	0.007227	CTRL-AHA; HPG-CTRL; MET-CTRL
745.4803	8.9494	0.001031	2.9867	0.007462	HPG-AHA; HPG-CTRL; MET-HPG
301.0027	8.9393	0.001037	2.9843	0.007488	HPG-AHA; HPG-CTRL; MET-HPG
439.0972	8.9284	0.001043	2.9817	0.007516	CTRL-AHA; HPG-CTRL; MET-HPG
235.0703	8.9246	0.001045	2.9808	0.007516	HPG-AHA; MET-HPG
455.2181	8.9212	0.001047	2.98	0.007516	HPG-CTRL; MET-HPG
252.9821	8.8973	0.001061	2.9744	0.007599	HPG-AHA; HPG-CTRL; MET-HPG
846.6278	8.8772	0.001072	2.9696	0.007645	HPG-CTRL; MET-HPG
774.5159	8.8768	0.001073	2.9696	0.007645	CTRL-AHA; HPG-CTRL; MET-CTRL
653.1026	8.8753	0.001074	2.9692	0.007645	HPG-AHA; HPG-CTRL; MET-HPG
824.6287	8.8683	0.001078	2.9675	0.007651	CTRL-AHA; HPG-AHA; MET-HPG
201.2283	8.8639	0.00108	2.9665	0.007651	CTRL-AHA; HPG-CTRL
550.4827	8.8629	0.001081	2.9663	0.007651	HPG-AHA; HPG-CTRL; MET-HPG
652.9641	8.8022	0.001117	2.9518	0.007894	CTRL-AHA; HPG-CTRL
179.9998	8.7849	0.001128	2.9477	0.007953	CTRL-AHA; HPG-CTRL; MET-HPG
289.0837	8.7759	0.001134	2.9456	0.007977	CTRL-AHA; HPG-CTRL
221.1447	8.7494	0.00115	2.9392	0.008078	CTRL-AHA; MET-AHA
187.0497	8.7293	0.001163	2.9344	0.008152	HPG-CTRL; MET-CTRL
123.0525	8.7121	0.001174	2.9303	0.008213	CTRL-AHA; HPG-CTRL; MET-CTRL
368.1138	8.7041	0.001179	2.9284	0.008234	HPG-AHA; HPG-CTRL; MET-HPG
717.5152	8.6849	0.001192	2.9238	0.008305	HPG-AHA; HPG-CTRL; MET-HPG
421.1276	8.6783	0.001196	2.9222	0.008319	HPG-AHA; HPG-CTRL; MET-HPG

273.116	8.671	0.001201	2.9205	0.008337	CTRL-AHA; MET-AHA; HPG-CTRL
322.9843	8.6607	0.001208	2.918	0.008368	HPG-AHA; HPG-CTRL; MET-HPG
330.0629	8.6462	0.001218	2.9145	0.008419	HPG-CTRL; MET-HPG
319.1122	8.6224	0.001234	2.9088	0.008515	HPG-CTRL; MET-HPG
551.4893	8.5941	0.001253	2.902	0.008619	HPG-AHA; HPG-CTRL; MET-HPG
629.4765	8.5936	0.001254	2.9018	0.008619	CTRL-AHA; HPG-CTRL; MET-HPG
205.0602	8.5482	0.001286	2.8909	0.008822	CTRL-AHA; HPG-CTRL; MET-HPG
163.0573	8.528	0.0013	2.886	0.008888	CTRL-AHA; HPG-CTRL; MET-CTRL
906.7182	8.525	0.001303	2.8852	0.008888	HPG-CTRL; MET-HPG
165.963	8.5247	0.001303	2.8852	0.008888	HPG-CTRL
110.0061	8.5158	0.001309	2.883	0.008906	CTRL-AHA; HPG-CTRL
146.0353	8.5137	0.001311	2.8825	0.008906	CTRL-AHA; MET-AHA; HPG-CTRL
251.0591	8.5095	0.001314	2.8815	0.008906	HPG-AHA; HPG-CTRL; MET-HPG
177.0467	8.5075	0.001315	2.881	0.008906	MET-AHA; HPG-CTRL; MET-HPG
466.1048	8.488	0.00133	2.8763	0.008969	CTRL-AHA; HPG-CTRL; MET-HPG
168.9857	8.4879	0.00133	2.8762	0.008969	CTRL-AHA; HPG-CTRL
702.0606	8.4838	0.001333	2.8752	0.008973	CTRL-AHA; HPG-CTRL; MET-HPG
310.1434	8.4734	0.001341	2.8727	0.009009	HPG-AHA; HPG-CTRL
244.0539	8.4627	0.001349	2.8701	0.009039	CTRL-AHA; HPG-CTRL; MET-CTRL
400.9482	8.4607	0.00135	2.8696	0.009039	CTRL-AHA; HPG-CTRL; MET-CTRL
388.0186	8.4192	0.001382	2.8595	0.009235	CTRL-AHA; HPG-CTRL; MET-HPG
391.1765	8.4074	0.001391	2.8566	0.009279	HPG-CTRL; MET-HPG
132.9808	8.3998	0.001397	2.8547	0.009302	CTRL-AHA; HPG-CTRL; MET-CTRL
159.0727	8.3676	0.001423	2.8469	0.009454	CTRL-AHA; HPG-CTRL
173.1975	8.3461	0.00144	2.8416	0.009552	CTRL-AHA; HPG-CTRL
244.0542	8.3403	0.001445	2.8402	0.009566	CTRL-AHA; HPG-CTRL; MET-CTRL
430.3803	8.2973	0.001481	2.8296	0.009784	CTRL-AHA; HPG-CTRL
202.1758	8.2828	0.001493	2.826	0.009834	CTRL-AHA; HPG-CTRL; MET-CTRL
199.0285	8.2815	0.001494	2.8257	0.009834	HPG-CTRL; MET-HPG
866.2809	8.2786	0.001496	2.825	0.009834	CTRL-AHA; HPG-CTRL; MET-HPG
325.0439	8.2618	0.001511	2.8208	0.009909	CTRL-AHA; HPG-CTRL; MET-CTRL
859.2768	8.2587	0.001513	2.8201	0.009909	CTRL-AHA; HPG-CTRL; MET-CTRL
779.4702	8.248	0.001523	2.8174	0.009951	CTRL-AHA; HPG-AHA
361.0966	8.2179	0.001549	2.81	0.010105	CTRL-AHA; HPG-CTRL; MET-CTRL
206.0442	8.2092	0.001557	2.8078	0.010137	CTRL-AHA; MET-AHA; HPG-CTRL
831.051	8.1978	0.001567	2.805	0.010172	MET-CTRL; MET-HPG
473.9481	8.1967	0.001568	2.8047	0.010172	CTRL-AHA; HPG-CTRL
240.1496	8.1697	0.001592	2.798	0.010311	CTRL-AHA; HPG-CTRL
860.9605	8.1616	0.0016	2.796	0.010341	CTRL-AHA; HPG-CTRL
376.9129	8.1445	0.001615	2.7917	0.010424	HPG-AHA; HPG-CTRL; MET-HPG
146.1617	8.141	0.001619	2.7909	0.010426	CTRL-AHA; HPG-CTRL; MET-CTRL
461.1197	8.111	0.001647	2.7834	0.010588	CTRL-AHA; HPG-AHA
274.119	8.106	0.001651	2.7822	0.010599	CTRL-AHA; MET-AHA; HPG-CTRL
690.141	8.0919	0.001665	2.7786	0.010666	HPG-AHA; HPG-CTRL; MET-HPG
629.226	8.0775	0.001679	2.775	0.010736	HPG-CTRL
124.0843	8.0717	0.001684	2.7736	0.010753	MET-AHA; MET-HPG
437.1762	8.0522	0.001703	2.7687	0.010855	HPG-CTRL; MET-HPG
527.8406	8.0457	0.00171	2.7671	0.010876	CTRL-AHA; HPG-CTRL; MET-CTRL
329.1277	8.0271	0.001728	2.7624	0.010974	CTRL-AHA; HPG-AHA; MET-AHA
361.1471	8.0103	0.001745	2.7582	0.011049	CTRL-AHA; HPG-CTRL
127.0115	8.0093	0.001746	2.7579	0.011049	HPG-CTRL; MET-HPG
116.0316	7.9943	0.001761	2.7541	0.011125	HPG-CTRL; MET-HPG
158.9182	7.9802	0.001776	2.7506	0.011197	CTRL-AHA; HPG-CTRL; MET-CTRL
466.1411	7.9747	0.001782	2.7492	0.011213	HPG-AHA; HPG-CTRL; MET-HPG
232.9253	7.9323	0.001826	2.7385	0.011472	HPG-AHA; HPG-CTRL; MET-HPG
490.808	7.9264	0.001832	2.737	0.011482	CTRL-AHA; HPG-CTRL; MET-CTRL
434.0106	7.9248	0.001834	2.7366	0.011482	HPG-AHA; MET-CTRL; MET-HPG
247.1262	7.9206	0.001838	2.7355	0.01149	HPG-AHA; HPG-CTRL; MET-HPG
429.1474	7.9073	0.001853	2.7322	0.011559	CTRL-AHA; MET-AHA
588.9201	7.8859	0.001876	2.7268	0.011672	HPG-AHA; HPG-CTRL; MET-HPG
262.1708	7.8827	0.00188	2.726	0.011672	CTRL-AHA; HPG-CTRL
356.9835	7.8817	0.001881	2.7257	0.011672	HPG-AHA; HPG-CTRL; MET-HPG
360.1437	7.8623	0.001902	2.7208	0.011785	CTRL-AHA; HPG-CTRL
487.0758	7.8512	0.001915	2.7179	0.011842	HPG-AHA; HPG-CTRL; MET-HPG
518.7544	7.8339	0.001934	2.7136	0.011941	CTRL-AHA; HPG-AHA; MET-HPG
430.1504	7.8215	0.001948	2.7104	0.012007	CTRL-AHA; MET-AHA
74.09463	7.8121	0.001959	2.708	0.012045	CTRL-AHA; HPG-CTRL
431.3857	7.8103	0.001961	2.7075	0.012045	CTRL-AHA; HPG-CTRL; MET-CTRL
343.9376	7.7958	0.001978	2.7038	0.012128	CTRL-AHA; HPG-CTRL
853.0327	7.7852	0.00199	2.7011	0.012159	MET-CTRL; MET-HPG
158.0433	7.7836	0.001992	2.7007	0.012159	CTRL-AHA; HPG-CTRL; MET-CTRL
296.0037	7.779	0.001997	2.6996	0.012159	CTRL-AHA; HPG-CTRL; MET-HPG
394.2301	7.7782	0.001998	2.6993	0.012159	CTRL-AHA; HPG-AHA
214.9126	7.7765	0.002	2.6989	0.012159	HPG-AHA; HPG-CTRL; MET-HPG

456.9886	7.774	0.002003	2.6983	0.012159	MET-AHA; MET-CTRL; MET-HPG
430.3806	7.7629	0.002016	2.6954	0.012202	CTRL-AHA; HPG-CTRL
293.0748	7.7622	0.002017	2.6952	0.012202	HPG-AHA; HPG-CTRL; MET-HPG
860.6283	7.7255	0.002061	2.6859	0.012448	CTRL-AHA; HPG-CTRL
204.0384	7.7145	0.002075	2.683	0.012505	CTRL-AHA; HPG-CTRL; MET-CTRL
209.0168	7.7121	0.002078	2.6824	0.012505	CTRL-AHA; HPG-CTRL; MET-CTRL
194.9619	7.6857	0.002111	2.6756	0.012667	HPG-AHA; HPG-CTRL; MET-HPG
566.7092	7.683	0.002114	2.6749	0.012667	CTRL-AHA; HPG-CTRL; MET-CTRL
425.1171	7.6818	0.002115	2.6746	0.012667	HPG-CTRL
134.9776	7.6617	0.002141	2.6695	0.012789	CTRL-AHA; HPG-CTRL; MET-CTRL
239.081	7.66	0.002143	2.669	0.012789	CTRL-AHA; MET-AHA; HPG-CTRL; MET-HPG
824.9636	7.6163	0.002199	2.6577	0.013092	HPG-AHA; MET-HPG
391.2776	7.615	0.002201	2.6574	0.013092	CTRL-AHA; HPG-CTRL; MET-HPG
323.1479	7.61	0.002208	2.6561	0.013109	CTRL-AHA; HPG-CTRL
412.0284	7.582	0.002245	2.6489	0.013308	CTRL-AHA; HPG-AHA; MET-CTRL
133.9249	7.554	0.002283	2.6416	0.01351	HPG-AHA; HPG-CTRL; MET-HPG
405.9614	7.5435	0.002297	2.6389	0.013573	CTRL-AHA; HPG-CTRL
59.05887	7.5298	0.002316	2.6353	0.013662	CTRL-AHA; HPG-CTRL
308.0853	7.5263	0.002321	2.6344	0.013668	HPG-CTRL; MET-HPG
194.0271	7.5045	0.002351	2.6287	0.013825	CTRL-AHA; MET-AHA
225.1553	7.4953	0.002364	2.6263	0.013879	CTRL-AHA; HPG-AHA; MET-AHA
104.0507	7.4657	0.002407	2.6186	0.014096	CTRL-AHA; MET-AHA; HPG-CTRL; MET-HPG
724.0422	7.4618	0.002412	2.6175	0.014096	HPG-CTRL; MET-HPG
142.0287	7.4614	0.002413	2.6174	0.014096	HPG-CTRL; MET-HPG
683.0647	7.4522	0.002426	2.615	0.014151	HPG-CTRL; MET-HPG
216.9555	7.4481	0.002432	2.614	0.014162	HPG-CTRL; MET-HPG
134.0414	7.433	0.002455	2.61	0.014269	MET-HPG
854.295	7.3411	0.002595	2.5859	0.015061	CTRL-AHA; HPG-CTRL
200.0322	7.3327	0.002608	2.5836	0.015113	CTRL-AHA; HPG-CTRL
724.9814	7.322	0.002625	2.5808	0.015187	HPG-AHA; HPG-CTRL; MET-HPG
314.1171	7.313	0.00264	2.5784	0.015234	CTRL-AHA; HPG-AHA; MET-AHA
513.0579	7.3117	0.002642	2.5781	0.015234	CTRL-AHA; HPG-AHA
300.2834	7.3074	0.002649	2.577	0.015249	CTRL-AHA; HPG-CTRL; MET-CTRL
871.2655	7.3043	0.002654	2.5761	0.015253	CTRL-AHA; HPG-CTRL
260.7554	7.2806	0.002692	2.5699	0.01545	CTRL-AHA; HPG-CTRL; MET-CTRL
428.3645	7.275	0.002702	2.5684	0.015479	CTRL-AHA; HPG-CTRL
395.152	7.2477	0.002747	2.5611	0.015692	HPG-AHA; HPG-CTRL; MET-HPG
543.9308	7.2473	0.002748	2.561	0.015692	HPG-AHA; HPG-CTRL; MET-HPG
290.9377	7.2383	0.002763	2.5586	0.015753	HPG-AHA; HPG-CTRL; MET-HPG
395.2331	7.231	0.002775	2.5567	0.015799	CTRL-AHA; HPG-AHA
291.9004	7.2199	0.002794	2.5537	0.015882	CTRL-AHA; HPG-CTRL
362.9962	7.2122	0.002808	2.5517	0.015897	CTRL-AHA; HPG-CTRL; MET-CTRL
866.9587	7.2114	0.002809	2.5515	0.015897	CTRL-AHA; HPG-CTRL
432.0961	7.2088	0.002813	2.5508	0.015897	CTRL-AHA; HPG-AHA; MET-AHA
531.7691	7.2052	0.00282	2.5498	0.015897	HPG-CTRL; MET-HPG
974.3374	7.2048	0.00282	2.5497	0.015897	CTRL-AHA; MET-AHA; HPG-CTRL
764.4486	7.2029	0.002824	2.5492	0.015897	CTRL-AHA; HPG-CTRL; MET-CTRL
573.8006	7.1893	0.002847	2.5456	0.016004	HPG-AHA; MET-HPG
206.0948	7.1613	0.002897	2.5381	0.016249	CTRL-AHA; HPG-CTRL
229.9019	7.1595	0.0029	2.5376	0.016249	CTRL-AHA; HPG-CTRL
172.9523	7.1376	0.002939	2.5318	0.01643	CTRL-AHA; MET-AHA; HPG-CTRL; MET-HPG
177.1199	7.1365	0.002941	2.5314	0.01643	MET-AHA; MET-HPG
867.957	7.1339	0.002946	2.5308	0.016431	CTRL-AHA; HPG-CTRL
765.5662	7.1218	0.002968	2.5275	0.016527	CTRL-AHA; HPG-CTRL
860.2959	7.1187	0.002974	2.5267	0.016527	CTRL-AHA; HPG-CTRL; MET-CTRL
309.128	7.1146	0.002981	2.5256	0.016527	HPG-AHA; HPG-CTRL; MET-HPG
230.1819	7.1144	0.002982	2.5255	0.016527	CTRL-AHA; HPG-AHA
160.0896	7.1065	0.002996	2.5234	0.016576	CTRL-AHA; HPG-CTRL
431.1484	7.1045	0.003	2.5229	0.016576	CTRL-AHA; MET-AHA; MET-HPG
715.4408	7.0583	0.003087	2.5104	0.017032	HPG-AHA; HPG-CTRL; MET-HPG
128.1403	7.0427	0.003117	2.5062	0.017171	CTRL-AHA; HPG-CTRL
807.2878	7.0365	0.003129	2.5046	0.01721	CTRL-AHA; MET-AHA; HPG-CTRL
619.0516	7.0231	0.003155	2.5009	0.01731	CTRL-AHA; HPG-CTRL; MET-CTRL
229.9018	7.0223	0.003157	2.5007	0.01731	CTRL-AHA; HPG-CTRL
867.6254	7.0167	0.003168	2.4992	0.017344	CTRL-AHA; HPG-CTRL; MET-CTRL
225.0264	7.0129	0.003176	2.4982	0.017358	MET-AHA; HPG-CTRL; MET-HPG
466.105	7.0025	0.003196	2.4954	0.017445	HPG-CTRL; MET-HPG
74.92914	6.9736	0.003254	2.4875	0.017735	CTRL-AHA; HPG-CTRL
358.0612	6.9681	0.003265	2.4861	0.017768	HPG-CTRL; MET-HPG
625.2765	6.9547	0.003293	2.4824	0.017873	CTRL-AHA; HPG-AHA; MET-HPG
858.6098	6.9539	0.003295	2.4822	0.017873	CTRL-AHA; HPG-CTRL; MET-HPG
429.9569	6.9459	0.003311	2.48	0.017935	CTRL-AHA; HPG-CTRL; MET-HPG
170.0081	6.9145	0.003377	2.4715	0.018263	CTRL-AHA; MET-AHA
430.0974	6.9109	0.003385	2.4705	0.018277	CTRL-AHA; HPG-AHA; MET-AHA

105.9511	6.8796	0.003452	2.462	0.01859	CTRL-AHA; HPG-CTRL
188.8759	6.8767	0.003458	2.4612	0.01859	CTRL-AHA; HPG-CTRL
687.0257	6.8767	0.003458	2.4612	0.01859	HPG-AHA; HPG-CTRL; MET-HPG
263.1549	6.8698	0.003473	2.4593	0.018634	CTRL-AHA; MET-AHA
175.1152	6.8681	0.003477	2.4588	0.018634	MET-HPG
172.1847	6.8655	0.003482	2.4581	0.018637	HPG-CTRL
338.9579	6.8398	0.003539	2.4511	0.018911	HPG-AHA; HPG-CTRL; MET-HPG
336.6841	6.8357	0.003549	2.45	0.018911	CTRL-AHA; HPG-CTRL; MET-CTRL
135.9737	6.8352	0.00355	2.4498	0.018911	CTRL-AHA; MET-AHA
243.0486	6.8062	0.003615	2.4419	0.019232	HPG-AHA; MET-HPG
589.5096	6.7923	0.003647	2.4381	0.019373	HPG-CTRL; MET-HPG
150.9762	6.7839	0.003667	2.4357	0.019448	CTRL-AHA; HPG-CTRL
176.0365	6.7742	0.003689	2.4331	0.019539	CTRL-AHA
851.2839	6.7667	0.003707	2.431	0.019603	CTRL-AHA; HPG-CTRL; MET-HPG
811.2944	6.7605	0.003721	2.4293	0.019651	HPG-AHA; HPG-CTRL; MET-HPG
608.078	6.7466	0.003754	2.4255	0.019797	CTRL-AHA; HPG-CTRL
132.115	6.7437	0.003761	2.4247	0.019802	HPG-AHA; HPG-CTRL
434.9663	6.74	0.00377	2.4236	0.019802	MET-CTRL; MET-HPG
146.044	6.7392	0.003772	2.4234	0.019802	CTRL-AHA; HPG-CTRL; MET-HPG
429.2329	6.733	0.003787	2.4217	0.019852	CTRL-AHA; MET-AHA; HPG-CTRL; MET-HPG
420.9916	6.7271	0.003801	2.4201	0.019897	HPG-CTRL; MET-HPG
671.06	6.7193	0.00382	2.4179	0.019942	HPG-CTRL; MET-HPG
454.9601	6.719	0.003821	2.4178	0.019942	HPG-CTRL
418.2052	6.6971	0.003875	2.4118	0.020192	MET-AHA; MET-CTRL; MET-HPG
134.1122	6.6858	0.003903	2.4086	0.020294	MET-AHA; MET-HPG
252.0098	6.6846	0.003906	2.4083	0.020294	CTRL-AHA; HPG-AHA
58.06368	6.6513	0.00399	2.3991	0.020701	CTRL-AHA; HPG-CTRL; MET-CTRL
429.233	6.6463	0.004002	2.3977	0.020737	CTRL-AHA; HPG-CTRL
253.0887	6.6434	0.00401	2.3969	0.020745	HPG-CTRL; MET-HPG
207.0727	6.6347	0.004032	2.3944	0.020832	CTRL-AHA; HPG-CTRL
168.071	6.6213	0.004067	2.3907	0.020982	HPG-CTRL; MET-HPG
177.0464	6.6161	0.004081	2.3893	0.020997	HPG-CTRL; MET-HPG
337.9747	6.6157	0.004082	2.3891	0.020997	CTRL-AHA; HPG-CTRL
825.9633	6.6079	0.004102	2.387	0.021071	HPG-CTRL; MET-HPG
225.1213	6.5839	0.004166	2.3803	0.021367	CTRL-AHA; HPG-CTRL
844.9418	6.5756	0.004188	2.378	0.021452	HPG-CTRL; MET-HPG
294.8486	6.5242	0.00433	2.3636	0.022143	CTRL-AHA; HPG-CTRL; MET-CTRL
323.0838	6.5126	0.004362	2.3603	0.022277	CTRL-AHA
119.0858	6.5077	0.004376	2.3589	0.02229	CTRL-AHA; HPG-CTRL; MET-CTRL
98.98167	6.5074	0.004377	2.3588	0.02229	HPG-CTRL
237.9869	6.4964	0.004408	2.3558	0.022417	CTRL-AHA
206.0625	6.4934	0.004417	2.3549	0.022428	CTRL-AHA; HPG-CTRL; MET-HPG
414.8051	6.4898	0.004427	2.3539	0.022449	CTRL-AHA; MET-CTRL
255.1402	6.4651	0.004499	2.3469	0.02278	HPG-AHA; MET-HPG
88.03718	6.4557	0.004526	2.3443	0.022886	MET-HPG
410.8482	6.4498	0.004543	2.3426	0.022942	CTRL-AHA; HPG-CTRL
538.7737	6.4131	0.004653	2.3322	0.023444	HPG-AHA; MET-HPG
825.6314	6.4122	0.004656	2.332	0.023444	HPG-AHA; HPG-CTRL; MET-HPG
124.0029	6.408	0.004669	2.3308	0.023475	CTRL-AHA; HPG-CTRL
379.1769	6.4037	0.004682	2.3296	0.023488	HPG-AHA; HPG-CTRL; MET-HPG
115.0916	6.3995	0.004695	2.3284	0.023488	HPG-AHA; HPG-CTRL
135.0445	6.3992	0.004696	2.3283	0.023488	HPG-CTRL; MET-HPG
334.1182	6.3986	0.004698	2.3281	0.023488	HPG-AHA; HPG-CTRL; MET-HPG
286.0806	6.3892	0.004727	2.3255	0.023599	CTRL-AHA; MET-AHA
871.5969	6.3862	0.004736	2.3246	0.023613	HPG-CTRL
74.09462	6.3814	0.004751	2.3233	0.023653	CTRL-AHA; HPG-CTRL
538.8754	6.3772	0.004764	2.322	0.023686	HPG-CTRL; MET-HPG
285.1212	6.3642	0.004805	2.3184	0.023856	CTRL-AHA; HPG-AHA; MET-AHA
286.1484	6.3367	0.004892	2.3105	0.024256	CTRL-AHA; HPG-AHA
808.2883	6.3344	0.004899	2.3099	0.024258	CTRL-AHA; HPG-CTRL
225.1195	6.3304	0.004912	2.3087	0.024288	CTRL-AHA; HPG-CTRL
550.2711	6.3247	0.00493	2.3071	0.024346	HPG-CTRL; MET-HPG
231.1849	6.3224	0.004938	2.3065	0.024349	CTRL-AHA; HPG-AHA; MET-AHA
269.1049	6.3126	0.00497	2.3037	0.024473	CTRL-AHA
85.0264	6.2957	0.005025	2.2988	0.024712	CTRL-AHA; HPG-CTRL
132.099	6.2884	0.00505	2.2967	0.024798	HPG-CTRL; MET-HPG
587.0269	6.2735	0.0051	2.2925	0.025008	CTRL-AHA; MET-AHA
304.136	6.2709	0.005108	2.2917	0.025015	HPG-CTRL; MET-HPG
578.9677	6.2672	0.005121	2.2907	0.025042	HPG-CTRL; MET-HPG
124.9612	6.2639	0.005132	2.2897	0.025063	HPG-CTRL; MET-HPG
448.1318	6.2571	0.005155	2.2878	0.025141	CTRL-AHA
105.9511	6.2415	0.005208	2.2833	0.025367	HPG-CTRL; MET-HPG
404.1343	6.2015	0.005348	2.2718	0.026013	CTRL-AHA; MET-AHA
236.0361	6.1694	0.005463	2.2625	0.026537	HPG-AHA; HPG-CTRL; MET-HPG

133.5581	6.1606	0.005495	2.26	0.026656	HPG-AHA; HPG-CTRL
845.959	6.1405	0.005569	2.2542	0.026979	HPG-AHA; MET-HPG
319.1924	6.1059	0.005699	2.2442	0.02754	HPG-AHA; HPG-CTRL; MET-HPG
331.0301	6.105	0.005703	2.2439	0.02754	HPG-CTRL; MET-HPG
402.1262	6.1036	0.005708	2.2435	0.02754	HPG-CTRL; MET-HPG
691.1376	6.085	0.00578	2.2381	0.027847	HPG-CTRL; MET-HPG
254.0082	6.0664	0.005852	2.2327	0.028157	CTRL-AHA; HPG-AHA
196.9568	6.062	0.005869	2.2314	0.028203	HPG-CTRL
808.7886	6.0434	0.005943	2.226	0.028518	CTRL-AHA; HPG-CTRL
173.0171	6.0409	0.005953	2.2253	0.028529	CTRL-AHA; HPG-CTRL
807.7887	6.0319	0.005989	2.2226	0.028662	CTRL-AHA; HPG-CTRL
132.099	6.021	0.006033	2.2195	0.028835	HPG-CTRL; MET-HPG
190.0086	6.0008	0.006116	2.2136	0.02919	CTRL-AHA; HPG-CTRL
424.1181	5.997	0.006131	2.2124	0.029203	HPG-AHA; MET-HPG
258.8941	5.9963	0.006135	2.2122	0.029203	HPG-CTRL
176.099	5.9929	0.006149	2.2112	0.02923	HPG-AHA; HPG-CTRL; MET-HPG
730.527	5.9873	0.006172	2.2096	0.029277	HPG-AHA; HPG-CTRL; MET-HPG
719.5306	5.9863	0.006176	2.2093	0.029277	HPG-CTRL; MET-HPG
401.952	5.9846	0.006183	2.2088	0.029277	HPG-AHA; HPG-CTRL; MET-HPG
222.1034	5.975	0.006223	2.206	0.029388	HPG-CTRL; MET-HPG
112.0188	5.9745	0.006225	2.2058	0.029388	HPG-AHA; HPG-CTRL
219.0232	5.9731	0.006231	2.2054	0.029388	CTRL-AHA; HPG-CTRL; MET-CTRL
866.6183	5.9618	0.006279	2.2021	0.029575	CTRL-AHA; HPG-CTRL
176.8903	5.9594	0.006289	2.2014	0.029584	CTRL-AHA; HPG-CTRL
290.1314	5.955	0.006308	2.2001	0.029633	MET-CTRL; MET-HPG
528.754	5.9517	0.006322	2.1991	0.02966	CTRL-AHA; HPG-CTRL
515.1381	5.9412	0.006367	2.1961	0.029833	CTRL-AHA; HPG-CTRL
338.6813	5.9277	0.006426	2.1921	0.030068	CTRL-AHA; HPG-CTRL
338.1318	5.9222	0.00645	2.1905	0.03012	HPG-AHA; HPG-CTRL; MET-HPG
712.2757	5.9213	0.006454	2.1902	0.03012	CTRL-AHA; HPG-CTRL
150.0708	5.9171	0.006472	2.189	0.030165	MET-AHA; MET-HPG
731.5305	5.9095	0.006505	2.1867	0.030262	HPG-AHA; MET-HPG
268.1257	5.9086	0.00651	2.1865	0.030262	CTRL-AHA; MET-AHA
168.0694	5.8985	0.006554	2.1835	0.03043	HPG-CTRL; MET-HPG
277.0398	5.8855	0.006613	2.1796	0.030661	CTRL-AHA; HPG-CTRL; MET-CTRL
143.0364	5.8834	0.006622	2.179	0.030665	HPG-AHA; HPG-CTRL
132.0992	5.8718	0.006675	2.1756	0.030869	CTRL-AHA; HPG-AHA
268.1029	5.8621	0.006719	2.1727	0.031032	CTRL-AHA; HPG-CTRL
428.3649	5.858	0.006737	2.1715	0.031078	CTRL-AHA; HPG-CTRL
187.1765	5.8493	0.006777	2.1689	0.031223	CTRL-AHA; MET-AHA
235.9886	5.8368	0.006836	2.1652	0.031451	CTRL-AHA; HPG-CTRL; MET-CTRL
362.9939	5.8336	0.006851	2.1643	0.031479	HPG-CTRL; MET-HPG
500.0828	5.8142	0.006942	2.1585	0.031828	HPG-CTRL
170.9538	5.8137	0.006944	2.1584	0.031828	HPG-CTRL; MET-HPG
272.0958	5.8033	0.006994	2.1553	0.032016	CTRL-AHA; HPG-AHA; MET-AHA
345.0416	5.7982	0.007019	2.1537	0.032087	HPG-CTRL; MET-HPG
344.7354	5.7954	0.007032	2.1529	0.032106	HPG-CTRL; MET-HPG
538.1633	5.7778	0.007117	2.1477	0.032454	CTRL-AHA
311.1792	5.769	0.00716	2.1451	0.03261	HPG-CTRL; MET-HPG
111.089	5.7651	0.007179	2.1439	0.032655	HPG-AHA; HPG-CTRL
771.5029	5.7595	0.007207	2.1422	0.032739	HPG-CTRL; MET-HPG
625.7776	5.7558	0.007226	2.1411	0.032756	HPG-AHA
734.8668	5.7551	0.007229	2.1409	0.032756	CTRL-AHA
514.135	5.7439	0.007285	2.1376	0.032966	CTRL-AHA; HPG-CTRL
865.2857	5.7367	0.007321	2.1354	0.033089	CTRL-AHA; HPG-CTRL
213.0117	5.733	0.00734	2.1343	0.033108	HPG-CTRL; MET-HPG
810.6258	5.7322	0.007344	2.1341	0.033108	HPG-AHA; MET-HPG
362.1484	5.7298	0.007356	2.1333	0.033122	HPG-CTRL
311.0342	5.7271	0.00737	2.1326	0.03314	CTRL-AHA; HPG-CTRL
482.2554	5.7246	0.007383	2.1318	0.033156	MET-HPG
638.1492	5.7056	0.00748	2.1261	0.033552	HPG-CTRL
405.1192	5.6865	0.007579	2.1204	0.033954	CTRL-AHA; HPG-CTRL
170.0774	5.6778	0.007625	2.1178	0.034117	CTRL-AHA; MET-AHA
720.5337	5.6597	0.007721	2.1123	0.034504	HPG-CTRL; MET-HPG
615.459	5.6502	0.007772	2.1095	0.034688	CTRL-AHA; HPG-CTRL
492.1201	5.6347	0.007857	2.1048	0.035022	CTRL-AHA; HPG-CTRL; MET-CTRL
381.1375	5.6258	0.007905	2.1021	0.035192	HPG-AHA; HPG-CTRL; MET-HPG
766.4085	5.6198	0.007938	2.1003	0.035271	HPG-AHA; MET-HPG
381.0165	5.619	0.007942	2.1001	0.035271	CTRL-AHA; HPG-CTRL
274.1349	5.6116	0.007984	2.0978	0.035412	HPG-AHA; HPG-CTRL; MET-HPG
140.0496	5.5969	0.008066	2.0934	0.03573	MET-HPG
524.049	5.5886	0.008113	2.0908	0.035858	CTRL-AHA; MET-CTRL
276.103	5.5882	0.008114	2.0907	0.035858	CTRL-AHA; HPG-CTRL
236.0651	5.5844	0.008136	2.0896	0.035885	CTRL-AHA; HPG-AHA

362.0085	5.5836	0.008141	2.0893	0.035885	HPG-CTRL; MET-HPG
342.2057	5.5768	0.00818	2.0873	0.036012	CTRL-AHA; MET-AHA
338.096	5.5705	0.008215	2.0854	0.036125	HPG-CTRL; MET-HPG
149.9895	5.5688	0.008225	2.0848	0.036125	HPG-CTRL
495.8513	5.566	0.008241	2.084	0.036151	CTRL-AHA; HPG-CTRL
410.9241	5.5629	0.00826	2.083	0.036186	HPG-CTRL; MET-HPG
375.0693	5.5601	0.008275	2.0822	0.036211	HPG-CTRL; MET-HPG
156.023	5.5567	0.008295	2.0812	0.036211	HPG-CTRL; MET-HPG
851.6175	5.5566	0.008296	2.0812	0.036211	HPG-CTRL
864.269	5.5541	0.00831	2.0804	0.03623	HPG-AHA; HPG-CTRL
701.4238	5.5453	0.008362	2.0777	0.036388	HPG-AHA; MET-HPG
347.1615	5.5444	0.008367	2.0774	0.036388	CTRL-AHA; HPG-CTRL
211.1435	5.5387	0.0084	2.0757	0.036489	MET-HPG
245.1557	5.5301	0.008451	2.0731	0.036665	HPG-AHA; HPG-CTRL
342.1334	5.5275	0.008466	2.0723	0.036687	CTRL-AHA; HPG-CTRL
586.3255	5.525	0.008481	2.0715	0.036708	MET-CTRL; MET-HPG
182.0407	5.5103	0.008569	2.0671	0.037043	HPG-CTRL; MET-HPG
318.1954	5.5071	0.008588	2.0661	0.037077	MET-AHA; MET-CTRL
145.0714	5.5055	0.008598	2.0656	0.037077	HPG-CTRL; MET-CTRL
718.527	5.497	0.008649	2.063	0.037254	HPG-CTRL; MET-HPG
536.264	5.4936	0.00867	2.062	0.0373	HPG-CTRL
210.1431	5.4867	0.008713	2.0599	0.037436	HPG-CTRL; MET-HPG
267.0563	5.4762	0.008777	2.0567	0.037608	HPG-AHA; MET-HPG
413.9823	5.4744	0.008788	2.0561	0.037608	CTRL-AHA; HPG-CTRL
136.9279	5.4739	0.008791	2.056	0.037608	CTRL-AHA; HPG-CTRL
231.8991	5.4734	0.008794	2.0558	0.037608	CTRL-AHA; HPG-CTRL
589.0331	5.4705	0.008812	2.0549	0.037637	CTRL-AHA; HPG-CTRL
439.0626	5.4673	0.008832	2.0539	0.037649	CTRL-AHA; HPG-CTRL
269.1444	5.4667	0.008836	2.0538	0.037649	HPG-AHA; MET-AHA
378.1742	5.4629	0.008859	2.0526	0.037705	HPG-CTRL; MET-CTRL
859.6124	5.4607	0.008873	2.0519	0.037719	HPG-CTRL
156.059	5.4503	0.008939	2.0487	0.037951	CTRL-AHA; MET-CTRL
743.3573	5.4472	0.008958	2.0478	0.037989	CTRL-AHA
393.1975	5.4322	0.009054	2.0432	0.038349	HPG-AHA; MET-HPG
402.3499	5.4256	0.009095	2.0412	0.03848	CTRL-AHA; HPG-CTRL
729.4556	5.4077	0.009211	2.0357	0.038924	HPG-AHA; MET-HPG
243.1	5.4004	0.009259	2.0334	0.039081	HPG-AHA; MET-AHA
113.1043	5.3781	0.009407	2.0266	0.039656	CTRL-AHA; HPG-CTRL
122.9214	5.3722	0.009446	2.0247	0.039733	HPG-CTRL
218.0622	5.3721	0.009447	2.0247	0.039733	HPG-CTRL
103.9531	5.3545	0.009566	2.0193	0.040175	HPG-CTRL
503.1652	5.3532	0.009575	2.0189	0.040175	CTRL-AHA; HPG-AHA
164.9262	5.3465	0.00962	2.0168	0.04032	CTRL-AHA
345.0149	5.3401	0.009664	2.0148	0.040457	CTRL-AHA; HPG-CTRL
777.4742	5.3291	0.009741	2.0114	0.040728	HPG-CTRL
282.1068	5.3238	0.009777	2.0098	0.040832	CTRL-AHA; HPG-AHA; MET-AHA
685.0449	5.3148	0.00984	2.007	0.041047	HPG-CTRL; MET-HPG
724.3783	5.3037	0.009918	2.0036	0.041325	CTRL-AHA; HPG-AHA
825.4211	5.2983	0.009957	2.0019	0.041438	HPG-AHA; MET-HPG
686.2203	5.2889	0.010024	1.999	0.041626	HPG-AHA; HPG-CTRL; MET-HPG
260.092	5.2887	0.010025	1.9989	0.041626	HPG-AHA; MET-AHA
292.1449	5.2828	0.010068	1.9971	0.041754	HPG-AHA; HPG-CTRL; MET-HPG
265.1069	5.2785	0.010099	1.9957	0.041833	CTRL-AHA; HPG-CTRL
169.0707	5.2723	0.010144	1.9938	0.041972	CTRL-AHA; MET-AHA
851.9508	5.2551	0.010269	1.9885	0.042443	HPG-CTRL; MET-HPG
214.0543	5.2461	0.010335	1.9857	0.042666	HPG-CTRL
161.1248	5.2372	0.010402	1.9829	0.042893	CTRL-AHA
260.0489	5.2308	0.01045	1.9809	0.043038	CTRL-AHA; HPG-CTRL; MET-CTRL
617.0556	5.2149	0.01057	1.9759	0.043485	CTRL-AHA; HPG-CTRL
159.0358	5.2091	0.010614	1.9741	0.043599	MET-HPG
290.1637	5.208	0.010622	1.9738	0.043599	HPG-AHA; HPG-CTRL
112.0479	5.1922	0.010745	1.9688	0.04405	CTRL-AHA; HPG-CTRL
435.0064	5.1904	0.010758	1.9683	0.044056	MET-HPG
278.8746	5.1862	0.010791	1.9669	0.044139	CTRL-AHA; MET-CTRL
449.1345	5.1769	0.010863	1.964	0.044385	CTRL-AHA
688.1451	5.1714	0.010907	1.9623	0.04448	HPG-CTRL
308.0426	5.1708	0.010912	1.9621	0.04448	HPG-CTRL
307.0599	5.1663	0.010947	1.9607	0.044574	CTRL-AHA
296.2165	5.1627	0.010976	1.9596	0.04464	CTRL-AHA; HPG-CTRL
649.2657	5.1573	0.011019	1.9579	0.044763	HPG-CTRL
744.2565	5.1462	0.011107	1.9544	0.045059	CTRL-AHA
88.03713	5.145	0.011117	1.954	0.045059	MET-AHA; MET-HPG
666.1204	5.141	0.011149	1.9528	0.045138	HPG-CTRL
834.5365	5.128	0.011254	1.9487	0.0455	HPG-CTRL

550.2256	5.1269	0.011264	1.9483	0.0455	CTRL-AHA; HPG-CTRL
182.9575	5.1203	0.011318	1.9462	0.045667	CTRL-AHA; HPG-CTRL; MET-CTRL
294.0544	5.0976	0.011506	1.9391	0.046372	HPG-CTRL; MET-HPG
151.9877	5.0869	0.011596	1.9357	0.046683	HPG-CTRL
244.8948	5.0784	0.011667	1.933	0.046919	HPG-CTRL
117.0516	5.0739	0.011705	1.9316	0.047019	HPG-CTRL
292.8122	5.0649	0.011782	1.9288	0.047274	HPG-CTRL
399.9637	5.0626	0.011802	1.928	0.047301	HPG-CTRL; MET-HPG
392.1779	5.06	0.011825	1.9272	0.047339	HPG-CTRL; MET-HPG
432.7755	5.042	0.011981	1.9215	0.047911	CTRL-AHA; HPG-CTRL; MET-CTRL
275.078	5.0397	0.012002	1.9208	0.04794	HPG-CTRL
280.1333	5.0272	0.012111	1.9168	0.048323	CTRL-AHA; HPG-CTRL
396.9908	5.0158	0.012213	1.9132	0.048674	HPG-CTRL
320.1855	5.005	0.01231	1.9097	0.048987	HPG-CTRL; MET-HPG
231.1291	5.004	0.012319	1.9094	0.048987	HPG-CTRL; MET-HPG
266.1098	5.0007	0.012349	1.9084	0.049053	CTRL-AHA
592.5292	4.9964	0.012387	1.907	0.049131	HPG-CTRL; MET-HPG
344.1288	4.9932	0.012416	1.906	0.049131	CTRL-AHA; HPG-CTRL
527.8404	4.9926	0.012422	1.9058	0.049131	CTRL-AHA; HPG-CTRL
323.0582	4.9924	0.012424	1.9058	0.049131	HPG-CTRL
872.948	4.9895	0.01245	1.9048	0.049181	CTRL-AHA; HPG-CTRL
178.1301	4.9735	0.012598	1.8997	0.049684	CTRL-AHA
499.1641	4.9727	0.012605	1.8995	0.049684	CTRL-AHA

Table S7. NMR metabolites.

Metabolite	Control		Met-50μM		AHA-50μM		HPG-50μM	
	Average	SD	Average	SD	Average	SD	Average	SD
2-Aminobutyrate	6.1	0.8	7.9	1.2	11.2	1.8	13	0.3
4-Aminobutyrate *	136.5	24.4	118.2	43.4	177.7	30.6	122.3	73.5
Acetate *	656.4	82.9	763.9	109.5	795.4	92.9	1001	90.7
Acetoacetate *	2.8	0.7	3.4	1.3	3.2	0.5	2.9	0.8
Acetoin *	3.8	3.4	3	1.2	2.5	0.8	5.1	1
Adenosine *	1.8	1.3	1.9	0.3	1.4	0.2	1.3	0.1
ADP	4.7	0.5	5.8	0.5	6.7	0.9	7.6	1.3
Alanine	178.7	36.8	208.9	22.4	288.3	52.9	344.5	39.6
AMP	25.9	3.5	33	3.2	43	8.7	62.7	3.9
Aspartate	18.3	7.2	23.2	12.3	13.6	1.6	18.5	1.6
ATP	2.6	0.9	4.7	1.2	5.7	1.4	6.2	1.7
Betaine	4.7	2.8	2.8	0.1	4	0.2	2.2	0.5
Cholate	2.4	1	2.6	0.5	3.1	0.7	3.7	0.7
Choline	1.5	1	1	0.1	1.3	0.2	1.3	0.1
Dimethyl sulfone *	1.2	1.3	0.8	0.3	0.7	0.1	1	0.2
Dimethylamine	0.5	0	0.7	0.2	0.8	0.1	1.3	0.3
dTTP *	23.4	1.9	26.6	2.5	35.5	5	29.6	3.9
Formate	382.5	50.2	678.9	36.6	705.6	69.4	642.7	16.6
Fumarate	6.5	1	5.8	0.9	4.8	0.9	11.2	2
Glucose	49	38.6	48.6	40.6	19.8	3.4	33.6	17.6
Glutamate	63.2	38.7	79.3	41.5	103.3	23.4	138.6	71.1
Glutathione	51.8	10.3	63.8	10.2	85.2	8.4	122.3	8.6
Glycine	50.4	15.8	57.8	11.1	104.2	10.4	81	5.5
GTP *	3.7	0.9	4.4	0.6	4.9	0.6	7.5	1.2
Histamine	2.4	0.7	2.1	0.5	3	1.1	3.9	0.6
Histidine	2.2	0.3	2.6	0.7	3.1	0.6	3.4	0.5
Hypoxanthine	17.2	5	23.1	1.4	27.4	4.5	26.8	5.8
IMP	3.4	1	5	0.8	5.6	1.7	7.1	0.3
Isoleucine	7.4	0.5	9.1	1	11.7	1.7	15.4	2.2
Lactate	53.9	21.1	104.5	43.4	130	40.1	123.3	24.3
Leucine	14.4	1.3	17.8	1.8	23	3.6	27.5	3.6
Malate	58.1	4.1	61.9	7.1	63.4	7.5	143.6	8.8
Methionine	6.7	1.3	8.3	1.2	13.3	1.5	14.2	1.4
N-Acetylaspartate *	54.2	12.7	96.9	5.6	101	10.9	61.2	12.9
N-Acetylglutamate *	5.9	1.1	6.1	1.3	7.2	0.5	11.7	2.5
N-Acetylglycine	3.5	0.4	3.6	0.8	4.4	0.5	6.6	0.7
NAD^+	15.1	0.7	21	3.4	27.2	4.5	30.4	3
N-Carbamoylaspartate *	83.3	25	117.7	25.4	128.4	10.2	61.9	17.9
O-Phosphocholine *	0.7	0.2	0.7	0.1	1	0.1	0.7	0.1
Pantothenate	3.6	1.8	4.6	0.7	2.9	1.1	4.8	1.6
Phenylalanine	5.5	0.6	5.9	0.5	8.1	1.1	10.3	1
Putrescine	5.9	1.4	7.2	0.7	11	2.1	16	1.6
Pyruvate	22.8	13.2	42.3	9.8	36.2	9.4	64.1	16.6
sn-Glycero-3-phosphocholine *	2	0.8	1.2	0.2	2.1	0.3	2.5	0.6
Succinate	152.9	19.3	205.9	16.2	259.5	34.6	316.5	50
Tartrate	6.6	0.9	8.2	1.8	10.4	1.3	12.8	1.4
Trimethylamine N-oxide	0.6	0.7	0.2	0.2	0.1	0.1	0.3	0.1
Tyrosine	12.7	2.1	17.6	1.8	22.4	2.8	25.5	3.8
UDP-glucose *	8.1	2.9	10.1	1.1	13.5	1.4	19.8	1.3
UDP-N-Acetylglucosamine *	3.1	1.1	2.8	1.8	2.8	0.6	3.5	0.6
UMP	20.4	2.8	19.7	1.4	26.8	3	35.7	1.2
Uracil	27.6	5.2	34.2	3	41.2	5.8	45.3	4.7
Valine	18.7	2.6	23.3	1.5	31.5	6.6	43.7	7.1
Xanthosine	1.4	0.3	1.9	0.2	1.4	0.3	1.3	0.2

*Assignment with best-matched signals, all others validated.

Table S8. Loading factors for 2D-PCA scores plot of heat-stressed *E. coli* cultures from Figure 3B.

Metabolite	PC1	PC2
2-Aminobutyrate	0.18327	-0.042002
4-Aminobutyrate	-0.012652	-0.18683
ADP	0.16284	-0.087807
AMP	0.17848	0.0011266
ATP	0.13204	-0.18101
Acetate	0.16316	-0.010254
Acetoacetate	0.015504	-0.14884
Acetoin	0.069363	0.17966
Adenosine	-0.0073455	0.018199
Alanine	0.17416	-0.029211
Aspartate	0.0020064	0.10747
Betaine	-0.069893	-0.0070998
Cholate	0.1413	0.059908
Choline	0.046082	0.16315
Dimethyl sulfone	0.029407	0.2601
Dimethylamine	0.15715	0.091441
Formate	0.12576	-0.26046
Fumarate	0.083996	0.29072
GTP	0.15447	0.014695
Glucose	-0.051666	0.053545
Glutamate	0.10733	0.089474
Glutathione	0.17402	0.030719
Glycine	0.12451	-0.14154
Histamine	0.13025	0.076048
Histidine	0.1491	0.0034941
Hypoxanthine	0.14492	-0.10708
IMP	0.16094	0.0042664
Isoleucine	0.18581	0.00074766
Lactate	0.13004	-0.124
Leucine	0.18495	-0.021526
Malate	0.15156	0.18264
Methionine	0.17547	-0.054267
N-Acetylaspartate	0.021105	-0.29746
N-Acetylglutamate	0.14634	0.18789
N-Acetylglycine	0.16222	0.079985
N-Carbamoylaspartate	-0.055414	-0.35668
NAD^+	0.17684	-0.083403
O-Phosphocholine	0.07488	-0.089337
Pantothenate	0.046712	0.11346
Phenylalanine	0.18293	0.04043
Propylene glycol	0.17549	-0.065088
Putrescine	0.18109	0.029436
Pyruvate	0.11508	-0.057185
Succinate	0.18301	-0.05308
Tartrate	0.16883	-0.0016467
Trimethylamine N-oxide	0.021261	0.27334
Tyrosine	0.17679	-0.090745

UDP-N-Acetylglucosamine	0.020713	0.12983
UDP-glucose	0.16733	-0.010497
UMP	0.16571	0.062401
Uracil	0.17514	-0.047694
Valine	0.18301	0.029837
Xanthosine	-0.059567	-0.14554
dTTP	0.12974	-0.18943
sn-Glycero-3-phosphocholine	0.093846	0.14058

**Table S9.** ANOVA of heat-stressed NMR metabolites.				
**#**	**Compound**	**f.value**	**p.value**	**-log10(p)**
1	Malate	100.3	1.38E-10	9.9
2	Formate	51.6	1.91E-08	7.7
3	UMP	41.8	8.46E-08	7.1
4	Putrescine	37.7	1.74E-07	6.8
5	Methionine	36.8	2.07E-07	6.7
6	Glutathione	35.2	2.83E-07	6.5
7	2-Aminobutyrate	34.7	3.10E-07	6.5
8	Phenylalanine	34.6	3.14E-07	6.5
9	AMP	34.5	3.24E-07	6.5
10	Isoleucine	32.2	5.18E-07	6.3
11	NAD^+	30	8.28E-07	6.1
12	Succinate	28.5	1.17E-06	5.9
13	Tyrosine	26.4	1.96E-06	5.7
14	Leucine	25.8	2.25E-06	5.6
15	Valine	24.1	3.53E-06	5.5
16	Fumarate	24	3.66E-06	5.4
17	Dimethylamine	22.4	5.64E-06	5.2
18	UDP-glucose	21.2	8.12E-06	5.1
19	N-Acetylaspartate	20.9	8.91E-06	5
20	N-Acetylglycine	19.9	1.18E-05	4.9
21	Tartrate	17.3	2.80E-05	4.6
22	Alanine	16.9	3.19E-05	4.5
23	N-Acetylglutamate	15.2	6.09E-05	4.2
24	GTP	15	6.58E-05	4.2
25	Uracil	13.7	1.08E-04	4
26	dTTP	11.5	2.82E-04	3.5
27	Glycine	11.5	2.84E-04	3.5
28	Acetate	11.2	3.25E-04	3.5
29	ADP	11.1	3.48E-04	3.5
30	IMP	10.4	4.90E-04	3.3
31	ATP	9.9	6.37E-04	3.2
32	N-Carbamoylaspartate	9.7	7.02E-04	3.2
33	sn-Glycero-3-phosphocholine	7.9	1.85E-03	2.7
34	Pyruvate	7.3	2.60E-03	2.6
35	Lactate	7.2	2.75E-03	2.6
36	Hypoxanthine	6.4	4.57E-03	2.3
37	Betaine	5	1.22E-02	1.9
38	Histidine	4.9	1.33E-02	1.9
39	Xanthosine	4.6	1.65E-02	1.8
40	Histamine	4.5	1.76E-02	1.8
41	O-Phosphocholine	3.7	3.38E-02	1.5

Table S10. Fold changes from NMR heat-stressed data.

Metabolite	Fold change		
	Met/Control	AHA/Control	HPG/Control
2-Aminobutyrate	1.3	1.9	2.2
4-Aminobutyrate	0.9	1.3	0.9
Acetate	1.2	1.2	1.5
Acetoacetate	1.2	1.1	1.0
Acetoin	0.8	0.6	1.3
Adenosine	1.1	0.8	0.7
ADP	1.2	1.4	1.6
Alanine	1.2	1.6	1.9
AMP	1.3	1.7	2.4
Aspartate	1.3	0.7	1.0
ATP	1.8	2.2	2.4
Betaine	0.6	0.9	0.5
Cholate	1.1	1.3	1.6
Choline	0.7	0.9	0.9
Dimethyl sulfone	0.6	0.6	0.8
Dimethylamine	1.2	1.4	2.3
dTTP	1.1	1.5	1.3
Formate	1.8	1.8	1.7
Fumarate	0.9	0.7	1.7
Glucose	1.0	0.4	0.7
Glutamate	1.3	1.6	2.2
Glutathione	1.2	1.6	2.4
Glycine	1.1	2.1	1.6
GTP	1.2	1.3	2.0
Histamine	0.9	1.2	1.6
Histidine	1.2	1.4	1.6
Hypoxanthine	1.3	1.6	1.6
IMP	1.5	1.6	2.1
Isoleucine	1.2	1.6	2.1
Lactate	1.9	2.4	2.3
Leucine	1.2	1.6	1.9
Malate	1.1	1.1	2.5
Methionine	1.3	2.0	2.1
N-Acetylaspartate	1.8	1.9	1.1
N-Acetylglutamate	1.0	1.2	2.0
N-Acetylglycine	1.0	1.3	1.9
NAD^+	1.4	1.8	2.0
N-Carbamoylaspartate	1.4	1.5	0.7
O-Phosphocholine	0.9	1.4	1.1
Pantothenate	1.3	0.8	1.3
Phenylalanine	1.1	1.5	1.9
Putrescine	1.2	1.9	2.7
Pyruvate	1.9	1.6	2.8
sn-Glycero-3-phosphocholine	0.6	1.0	1.3
Succinate	1.3	1.7	2.1
Tartrate	1.2	1.6	1.9
Trimethylamine N-oxide	0.4	0.2	0.6

Tyrosine	1.4	1.8	2.0
UDP-glucose	1.3	1.7	2.5
UDP-N-Acetylglucosamine	0.9	0.9	1.1
UMP	1.0	1.3	1.8
Uracil	1.2	1.5	1.6
Valine	1.2	1.7	2.3
Xanthosine	1.4	1.0	0.9
