# Supplementary figures and images for: Metabolic Implications of Using BioOrthogonal Non-Canonical Amino Acid Tagging (BONCAT) for Tracking Protein Synthesis
Source: Front Microbiol. 2020 Feb 13;11:197. doi: 10.3389/fmicb.2020.00197 (PMC7031258; doi:10.3389/fmicb.2020.00197)

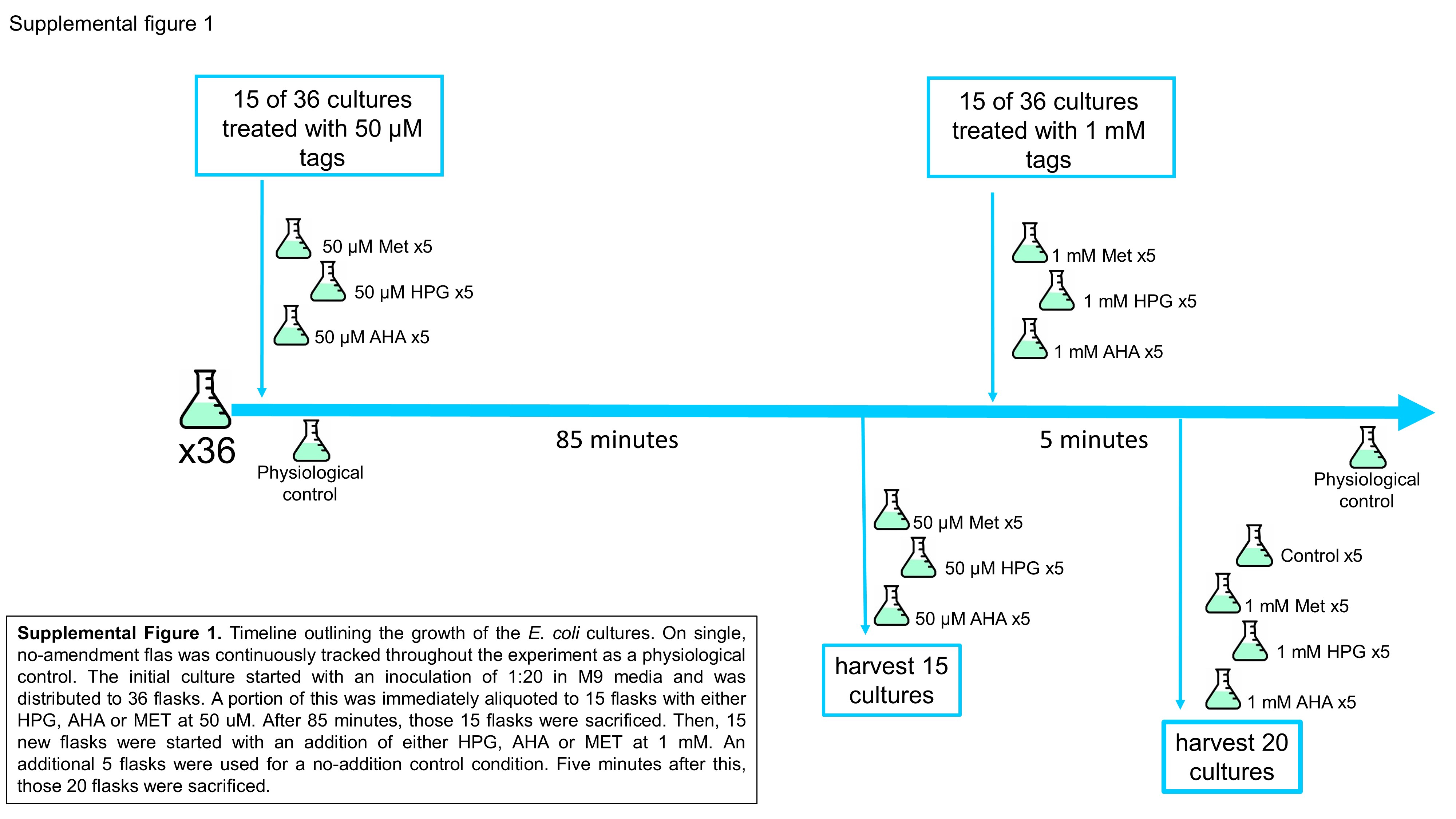

Supplement: Supplementary file 2 [file Image_1.jpg]

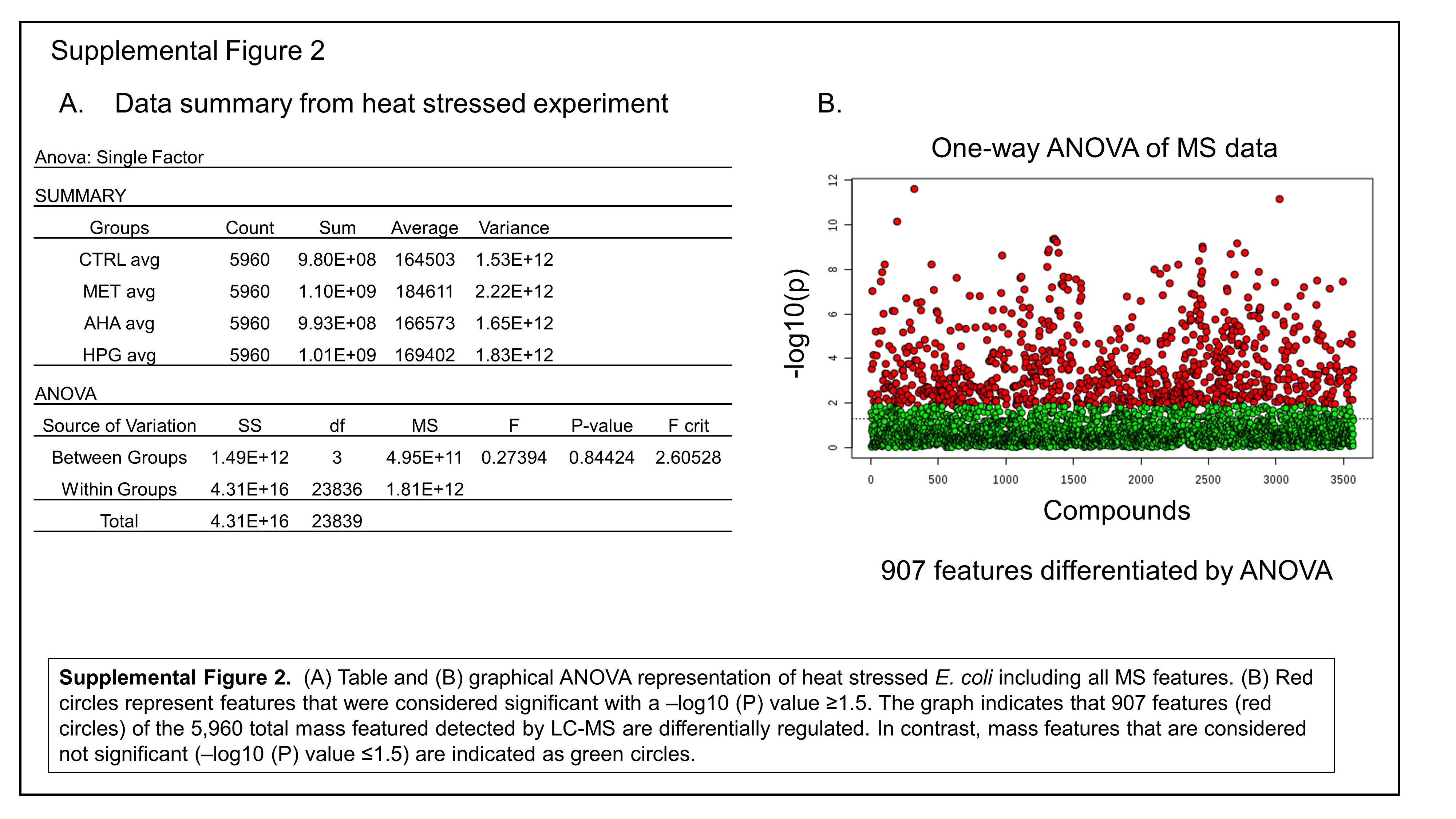

Supplement: Supplementary file 3 [file Image_2.jpg]

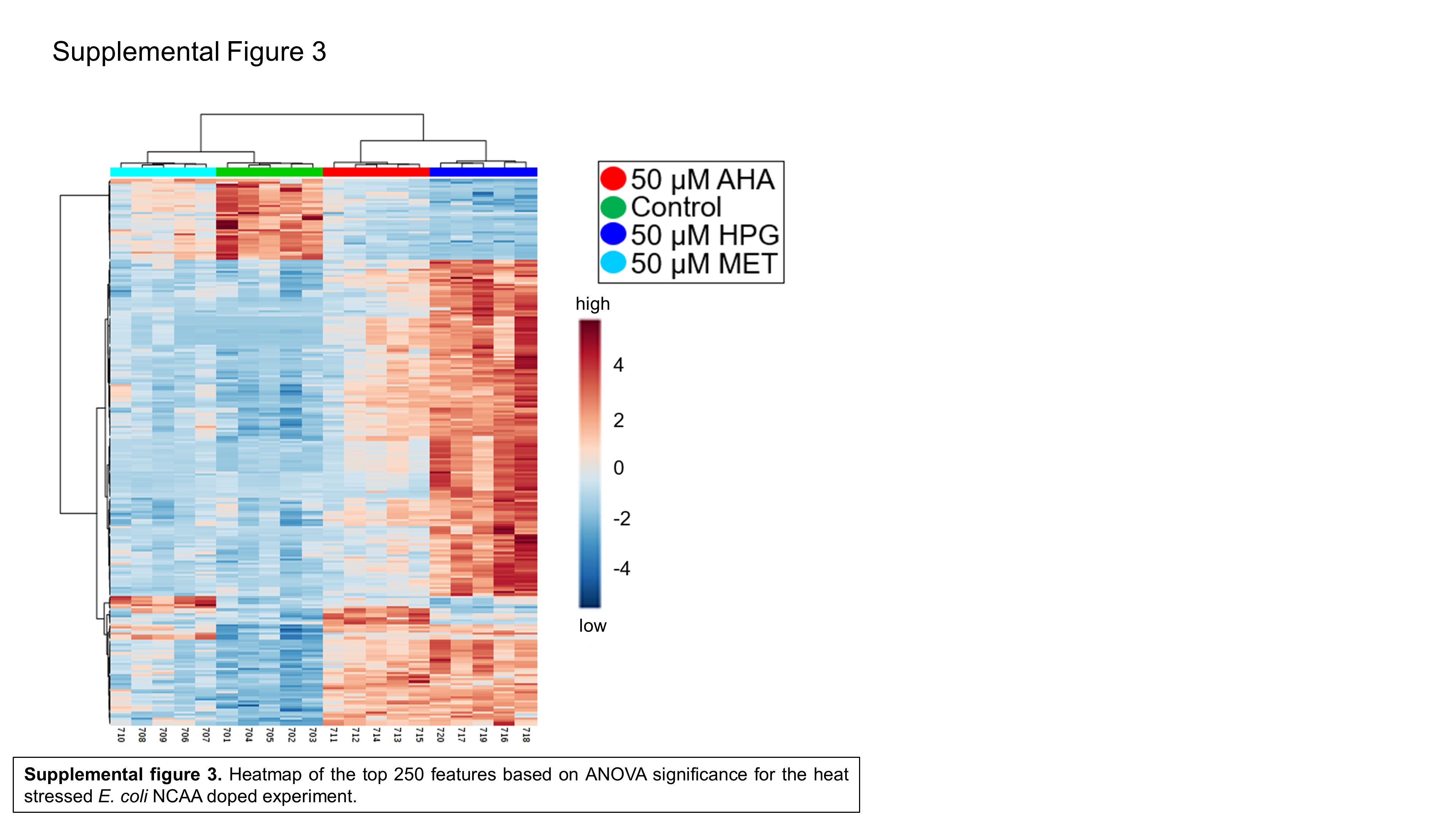

Supplement: Supplementary file 4 [file Image_3.jpg]

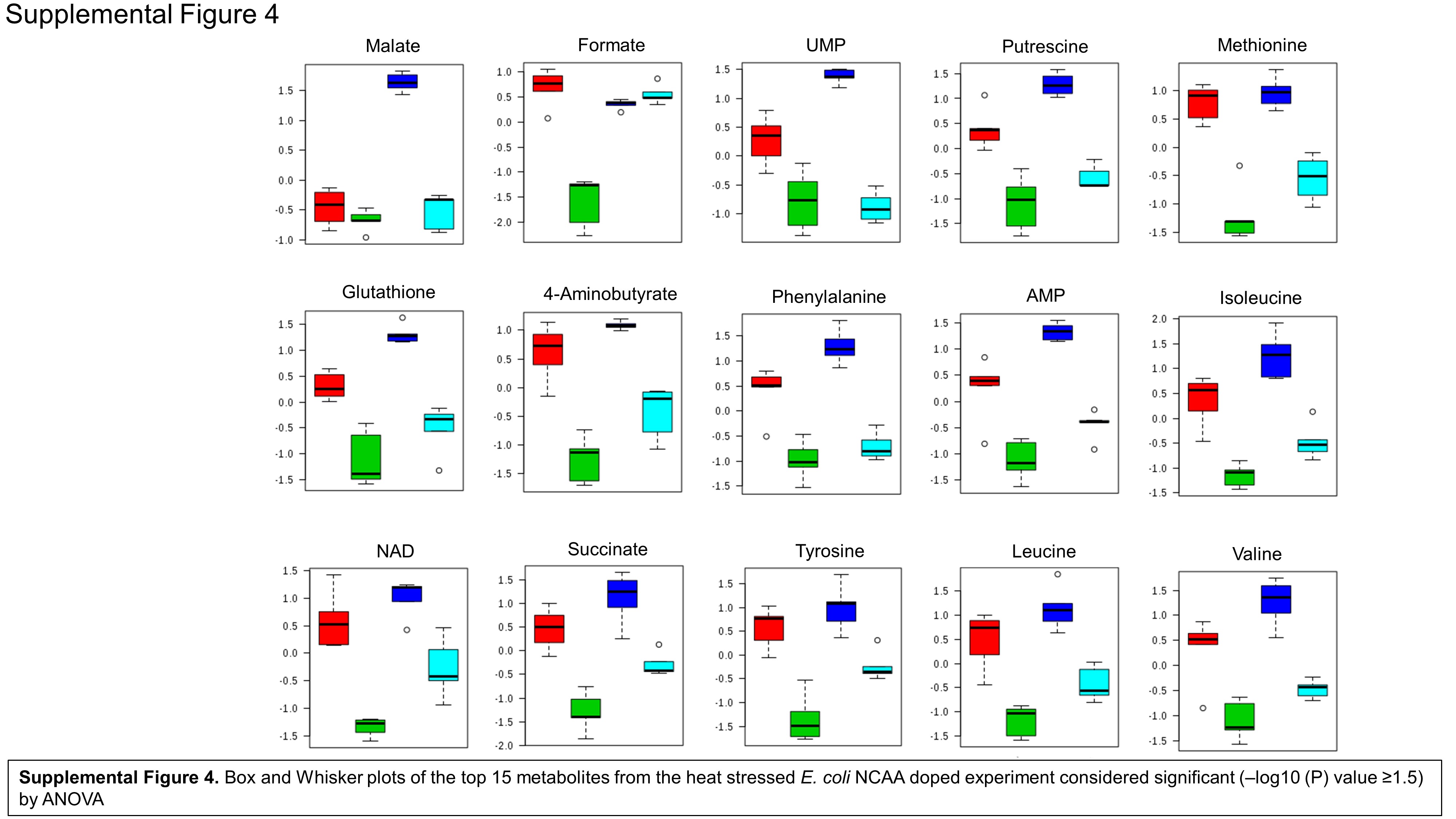

Supplement: Supplementary file 5 [file Image_4.jpg]

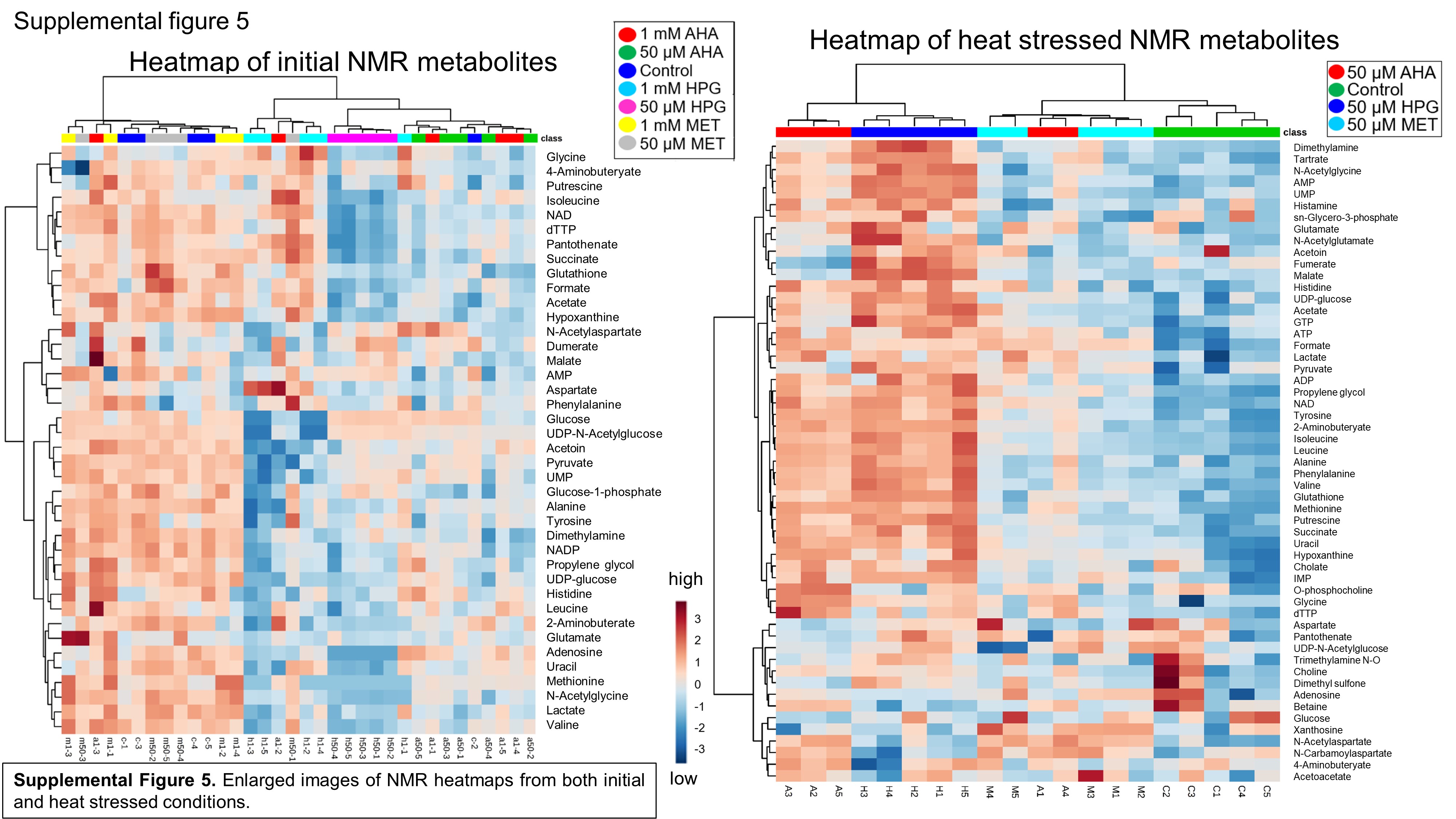

Supplement: Supplementary file 6 [file Image_5.jpg]
